# A systems genomics approach to uncover patient-specific pathogenic pathways and proteins in ulcerative colitis

**DOI:** 10.1038/s41467-022-29998-8

**Published:** 2022-04-28

**Authors:** Johanne Brooks-Warburton, Dezso Modos, Padhmanand Sudhakar, Matthew Madgwick, John P. Thomas, Balazs Bohar, David Fazekas, Azedine Zoufir, Orsolya Kapuy, Mate Szalay-Beko, Bram Verstockt, Lindsay J. Hall, Alastair Watson, Mark Tremelling, Miles Parkes, Severine Vermeire, Andreas Bender, Simon R. Carding, Tamas Korcsmaros

**Affiliations:** 1 Earlham Institute, Norwich Research Park, Norwich, UK; 2 Gut Microbes and Health Programme, The Quadram Institute Bioscience, Norwich Research Park, Norwich, UK; 3 Department of Clinical, Pharmaceutical and Biological Sciences, University of Hertfordshire, Hertford, UK; 4 Gastroenterology Department, Lister Hospital, Stevenage, UK; 5 Centre for Molecular Science Informatics, Department of Chemistry, University of Cambridge, Cambridge, UK; 6 KU Leuven, Department of Chronic diseases, Metabolism and Ageing, Leuven, Belgium; 7 Department of Gastroenterology, Norfolk and Norwich University Hospitals, Norwich, UK; 8 Department of Genetics, Eötvös Loránd University, Budapest, Hungary; 9 Department of Molecular Biology, Semmelweis University, Budapest, Hungary; 10 University Hospitals Leuven, Department of Gastroenterology and Hepatology, KU Leuven Leuven, Belgium; 11 Norwich Medical School, University of East Anglia, Norwich, UK; 12 School of Life Sciences, ZIEL - Institute for Food & Health, Technical University of Munich, 80333 Freising, Germany; 13 Inflammatory Bowel Disease Research Group, Addenbrooke’s Hospital, University of Cambridge, Cambridge, UK

**Keywords:** Systems biology, Ulcerative colitis

## Abstract

We describe a precision medicine workflow, the integrated single nucleotide polymorphism network platform (iSNP), designed to determine the mechanisms by which SNPs affect cellular regulatory networks, and how SNP co-occurrences contribute to disease pathogenesis in ulcerative colitis (UC). Using SNP profiles of 378 UC patients we map the regulatory effects of the SNPs to a human signalling network containing protein-protein, miRNA-mRNA and transcription factor binding interactions. With unsupervised clustering algorithms we group these patient-specific networks into four distinct clusters driven by PRKCB, HLA, SNAI1/CEBPB/PTPN1 and VEGFA/XPO5/POLH hubs. The pathway analysis identifies calcium homeostasis, wound healing and cell motility as key processes in UC pathogenesis. Using transcriptomic data from an independent patient cohort, with three complementary validation approaches focusing on the SNP-affected genes, the patient specific modules and affected functions, we confirm the regulatory impact of non-coding SNPs. iSNP identified regulatory effects for disease-associated non-coding SNPs, and by predicting the patient-specific pathogenic processes, we propose a systems-level way to stratify patients.

## Abstract (web summary)

Single Nucleotide Polymorphisms (SNPs) affect cellular regulatory networks, and SNP co-occurrences contribute to disease pathogenesis in ulcerative colitis (UC). Here the authors introduce iSNP, a precision medicine pipeline that combines genomics and network biology approaches to uncover patient specific pathways affected in complex diseases.

## Introduction

Precision medicine is a key clinical goal for the effective treatment of heterogeneous, complex diseases such as inflammatory bowel disease (IBD). Complex, multilayered, integrative techniques are required to identify the individual patients’ complex pathogenic pathways^1,2^. With IBD, the interlinked facets leading to disease are a dysfunctional immune system and response to environmental triggers, including constituents of the intestinal microbiota and dietary factors, in a genetically susceptible host^3^. Focusing solely on genetic susceptibility, genome-wide association studies (GWAS) and subsequent fine mapping of identified regions defined causal disease-associated single nucleotide polymorphisms (SNPs)^4,5^. However, the clinical impact of these SNPs has yet to be realised. A promising approach to assess the functional role of SNPs, and advise clinical practice, is to examine patient-specific sets in combination with systems-level approaches^6^.

Exome sequencing and protein structural biology have already contributed to the functional annotation of SNPs in protein-coding regions (that alter the amino acid composition and the function of the translated proteins), and how they impact diseases such as obesity^7^, IBD^5^ and lung cancer^8^. Computational workflows prioritise such coding SNPs for further analysis^9^. These approaches include artificial intelligence methodologies (such as machine learning and deep neural networks) to identify and quantify deleterious regulatory impacts of SNPs using chromatin accessibility and transcription factor binding affinities^10^, and high-throughput chromatin interaction studies^11^. This allows for the identification of SNPs of interest. However, understanding the function of SNPs in non-coding regions of the DNA remains challenging, principally because many disease-causing SNPs are in areas yet to be annotated^5^.

In ulcerative colitis (UC), a form of IBD, coding SNPs comprises less than 10% of the total UC-associated SNPs^12^. These coding SNPs are not causally related to impaired intestinal barrier function or inflammation that are hallmark pathognomic features of UC^13^. Understanding of the phenotypic effects of SNPs in IBD has involved the study of monogenic IBD in paediatrics that analysed the deleterious nature of non-coding SNPs^14^, although in adult-onset IBD these rare individual phenotypic SNPs have not been identified^15^. A broader and deeper understanding of the function of SNPs in this complex genetic disease is therefore needed.

We propose that functional annotation at the molecular and systems-level of the remaining 90% SNPs located in non-coding regions would expand the utility of these disease-associated SNPs. The proposed gap-filling systems-level analysis is essential, as individual SNPs may have subtle phenotypic effects, but in combination, they may have a pathological impact. Integrated analysis of these non-coding SNPs allows the identification of novel pathogenic pathways, and potentially patient-specific disease susceptibility, thus facilitating precision therapy.

For functional annotation of SNPs in non-coding regions, a key question is whether the SNPs affect gene expression by, for example, affecting long non-coding RNAs^16–20^, microRNA-target sites (miRNA-TS)^21^, splicing^22–25^ or transcription factor (TF) binding sites (TFBS)^26^ in promoter regions and within the first introns^27^, which has been reported in complex diseases such as diabetes, schizophrenia, coronary heart disease and Crohn’s disease^28–31^. In this study, we focused on two regulatory effects as examples; SNPs occurring in transcription factors binding sites and in miRNA target sites as they can be validated by published studies.

To identify the effect of non-coding SNPs, we have built on the concepts identified by Boyle et al. to track the cumulative effects of multiple regulatory SNPs as an ‘omnigenic’ model^32^. Using network biology approaches that we have previously exploited to uncover novel and important proteins in cancer biology^33^, we aimed to further understand the pathogenic pathways of UC and to identify novel and previously hidden disease-associated proteins. These proteins are often undetected or hidden in conventional mutation and expression screens as they mostly act as direct interactors (first neighbours) of the proteins affected by a disease-associated SNP. Using first neighbours gives an optimal trade-off to keep specificity while reconstructing a connected graph. Similar studies have utilised the concept of first neighbour proteins in both type 2 diabetes^34^ and juvenile idiopathic arthritis^35^. Systems biology approaches have been utilised with predictive network models that identified proteins involved in the pathogenesis of IBD in general^36–38^ but these approaches are unable to account for regulatory and downstream effects of non-coding SNPs. Therefore, by identifying first neighbour proteins in UC, we aimed to expand current research and identify additional pathogenic pathways of pharmacological use in UC that have been previously undetected or hidden due to a lack of connection with non-coding UC-associated SNPs. As UC is highly heterogeneous, we used individual patient data to identify patient cohorts with similar or different pathogenic pathways of UC.

Connecting non-coding SNPs to pathways, especially in a patient-specific manner, is a much needed but highly challenging approach. To achieve this, we developed a workflow, named the integrative SNP Network Platform (iSNP) by combining systems genomics and network biology approaches into a scalable system. We demonstrated its applicability by analysing a UC-associated signalling network and by identifying patient clusters with distinct pathomechanisms contributing to UC. Within these clusters, we highlighted cluster-specific key players, identifying known and additional proteins as well as patient-specific pathways to the disease. These predicted pathogenic effects were then validated using transcriptomic data from an independent patient cohort^39^. Integrating systems genomics and network biology data and analysis offers unique biological insights and enables the scalable examination of patient-specific datasets for precision medicine.

## Results

### Constructing the UC-associated signalling network

**Fig. 1:**
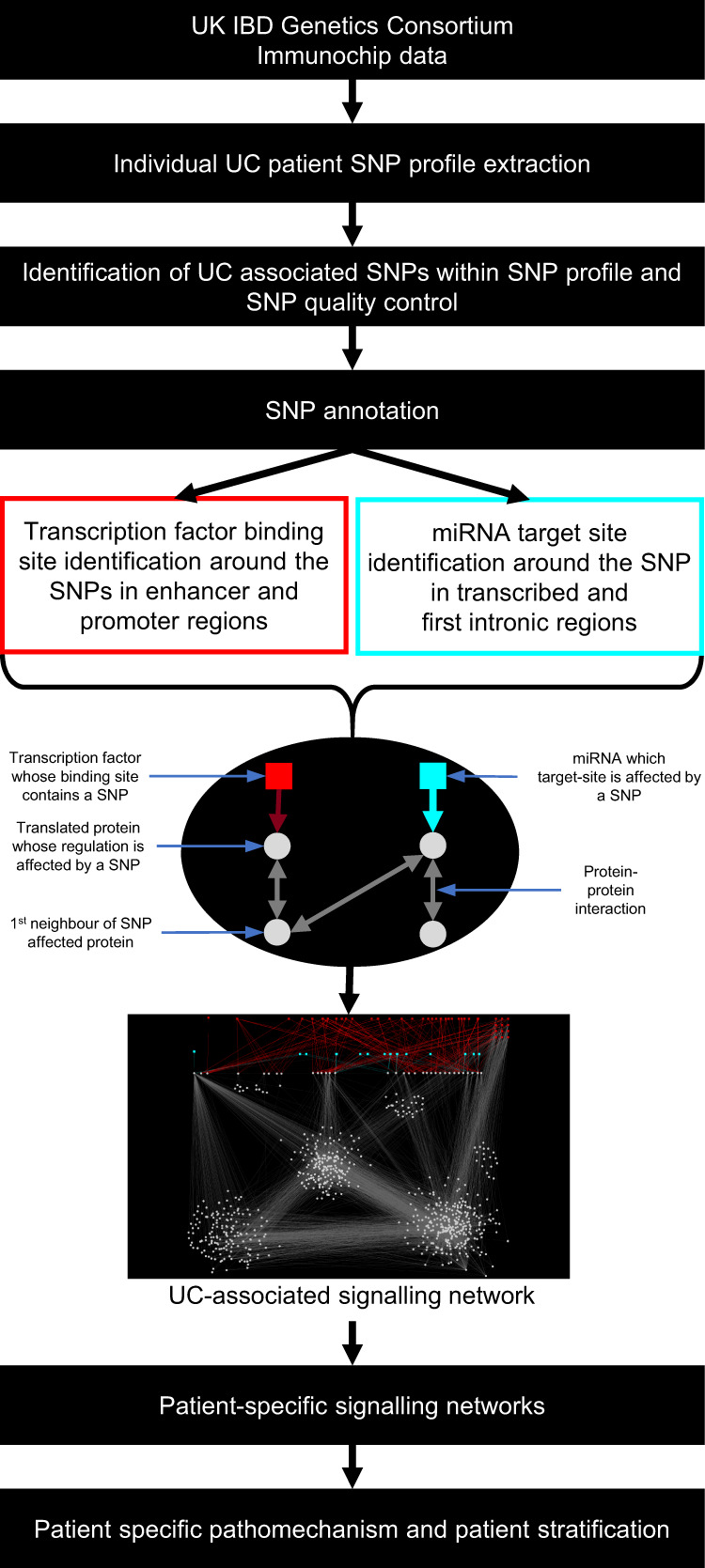
The iSNP workflow and its application to reconstruct a ulcerative colitis-associated signalling network for non-coding single nucleotide polymorphisms. Single nucleotide polymorphisms (SNP) identified in patients were annotated based on those occurring within transcription factor binding sites (TFBS) localised in enhancer or promoter regions of genes, or within microRNA-target sites (miRNA-TS) that are in first intronic regions or untranslated regions. After identifying the proteins whose transcription or translation could be affected by these non-coding SNPs, their protein interactors (first neighbours) were determined to construct a ulcerative colitis-associated signalling network. (UK: United Kingdom, IBD: Inflammatory Bowel Disease).

The integrative SNP Network Platform (iSNP) was developed to assess the regulatory effects of non-coding SNPs. The iSNP workflow constructs an integrated network based on identifying the proteins whose expression could be directly affected by the SNPs (termed as SNP-affected proteins) and their interactors (first neighbours) through protein–protein interactions. (Fig. 1, Supplementary Fig. 1). We used the UK East Anglia cohort of 378 patients from the UK IBD genetics consortium for this analysis.

Patients from this cohort had a total of 40 individual UC-associated SNPs from which we identified 22 UC-associated regulatory SNPs localised within TFBS or miRNA-TS. These SNPs were annotated to occur within 20 TFBSs and 4 individual miRNA-TSs (Table 1, Supplementary Data 1). 11 of the affected TFBSs were in enhancer regions and 3 were in both enhancer and promoter regions. Each of this affected TFBS and miRNA-TS has multiple TFs and miRNAs binding to them resulting in 264 transcription factors and 405 miRNAs whose regulatory function is affected by these non-coding SNPs (Supplementary Data 1). These regulators are involved in a total of 1490 regulatory interactions (923 TF-TFBS and 550 miRNA-miRNA-TS interactions). The identified regulatory interactions affected by the non-coding SNPs led us to determine the genes whose expression could be impacted by a SNP. These regulatory interactions potentially affected 48 genes.

**Table 1:** Affected SNPs in the UC-associated signalling network, their target genes and interactions^a^.

SNP	Target gene name	Regulatory annotation of the SNP
rs11041476	*LSP1*	TFBS in an enhancer, miRNA-TS in the first intron
	*TNNI2*	TFBS in an enhancer
rs11168249	*RAPGEF3*	TFBS in an enhancer
	*HDAC7*	TFBS in an enhancer, miRNA-TS in the first intron
rs11676348	*ARPC2*	TFBS in an enhancer
	*CXCR1*	
	*CXCR2*	
	*SLC11A1*	
	*CTDSP1*	
rs12254167	*CCNY*	TFBS in an enhancer
rs1598859	*NFKB1*	TFBS in an enhancer
	*CISD2*	
rs17085007	*RPL21*	TFBS in an enhancer
	*GTF3A*	
rs1801274	*FCGR2A*	miRNA-TS in an exon
rs3774937	*NFKB1*	miRNA-TS in an intron
**rs477515**	***HLA-DQA2***	**TFBS in an enhancer**
	***HLA-DQB1***	
	***HLA-DQB2***	
	***C4A***	
	***HSPA1B***	
	***HLA-DPA1***	
	***AGER***	
	***NOTCH4***	
rs543104	*CCDC82*	TFBS in an enhancer
rs559928	*RPS6KA4*	TFBS in an enhancer
rs6087990	*DNMT3B*	TFBS in a promoter
**rs7404095**	***PRKCB***	**miRNA-TS in a intron**
rs907611	*LSP1*	TFBS in a promoter
**rs913678**	***SNAI1***	**TFBS in an enhancer**
	***CEBPB***	
	***PTPN1***	
**rs943072**	***VEGFA***	**TFBS in an enhancer**
	***XPO5***	
	***POLH***	

^a^Details of each interaction are provided in Supplementary Table 1. Cluster-driving SNPs affecting the regulation of a high number of proteins directly or through their first neighbours are shown in bold.

**Fig. 2:**
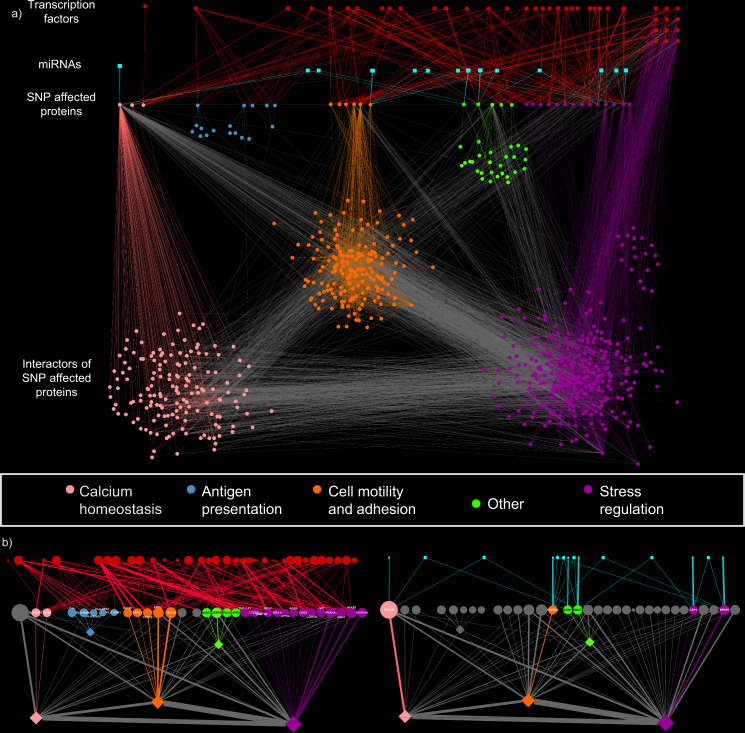
Visualisation and modularisation of the ulcerative colitis-associated signalling network. **a** The ulcerative colitis (UC)-associated signalling network contains proteins affected by—associated single nucleotide polymorphisms (SNPs), their interactor partners as well as the transcription factors(TF) and micro-RNAs(miRNA) whose binding or target sites are affected by a SNP. Circles represent proteins and squares represent regulators (red = TFs, blue = miRNAs). Nodes are coloured according to network modules. The modules are named by their representative function. At the top right side of the network are TFs involved in potential regulatory feedback loops in UC pathogenesis. **b** Visualisation of the two regulatory modules. The module on the left represents the transcription factor binding sites based effects on the downstream network, which affects almost the entire signalling network. The module on the right represents the microRNA-target site based effects that mainly happen by regulating *PRKCB*.

The products of the genes predicted to be affected by the SNPs were filtered for proteins present in the OmniPath network, an integrated and comprehensive resource for manually curated signalling interaction databases. Of the 48 SNP-affected proteins, 33 were in the OmniPath network^40,41^ and were regulated by 169 TFs and 247 miRNAs. To uncover the larger effect space of the non-coding SNPs, we identified the first neighbour interactors of the 33 SNP-affected proteins. In total, the UC-associated signalling network consisted of 686 protein nodes, 6808 protein–protein interactions resulting in 758 regulatory interactions (Fig. 2a).

The UC-associated signalling network contains three major parts or modules, each over-represented with functions relevant in UC: (1) calcium homeostasis; (2) cell motility and adhesion; (3) stress regulation. Two additional modules were identified, one containing HLA receptors involved in antigen-presentation and one containing other proteins such as MAPKs or HDAC7.

The network visualisation shown in Fig. 2 highlights the weighting of each SNP in the iSNP workflow; if a single nucleotide polymorphism is in a miRNA-TS, enhancer or promoter of a hub protein, which has a high number of neighbours, then it has a larger effect on the network compared to other proteins. This is particularly apparent for the two main SNPs that are the driving force behind the constructed network: rs7404095 and rs913678. rs7404095 affects *PRKCB* gene through a miRNA -TS whereas rs913678 affects *PTPN1*, *CEBPB* and *SNAI1* genes through a TFBS in an enhancer region.

The UC-associated signalling network uncovered interesting regulatory feedback loops (Fig. 2a). In these loops, TFs (Fig. 2a, listed in Supplementary Data 2A) are regulatory genes encoding proteins that interact with the same TF at the protein–protein level. The TFs include key stress response regulators, such as MYC, JUN, PPARA, PPARG, CEBPA and HIF1A. By using the whole feedback loop for a Gene Ontology biological process enrichment test, they were enriched in relation to cell proliferation, wound healing, angiogenesis regulation, stress response and cytokine response (Supplementary Data 2C). Thus, these feedback loops are affected by UC-associated regulatory SNPs and systematically perturb cellular processes critical in UC pathogenesis.

### Identification of patient-specific clusters based on the UC-associated network

**Fig. 3:**
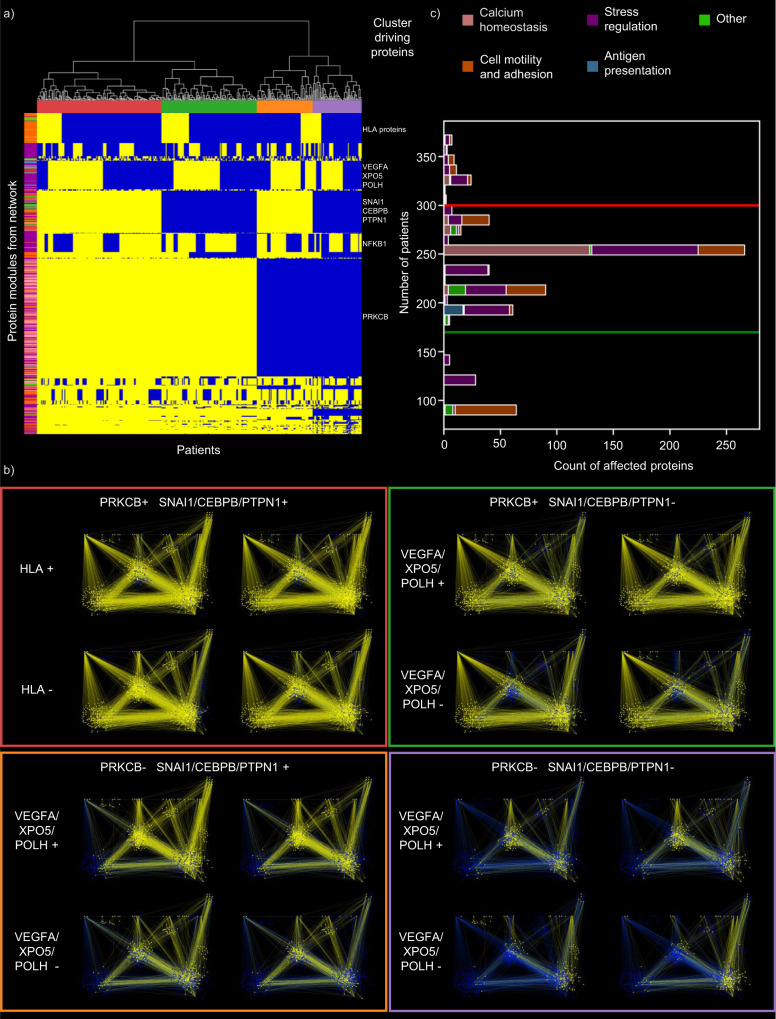
Unsupervised clustering of ulcerative colitis patients based on their network footprint. **a** Heatmap of directly or indirectly affected proteins in each patient. Each column represents a patient, and each row is a protein. Yellow colouring indicates specific proteins affected in individual patients while blue means the opposite. The hierarchical clustering of the patients is shown above the heatmap and was generated using Hamming distance with the average clustering method in which colours represent the patient clusters. The left of the heatmap identifies the proteins in various patient-specific modules, while cluster-driving proteins are shown on the right side of the heatmap. **b** Representative networks from the four patient clusters. Yellow colour indicates directly or indirectly affected proteins, while blue colour indicates not affected proteins. **c** Histograms depict the number of patients in which a given protein is affected. The horizontal red line demarcates affected proteins in more than 300 patients. The green line defines the cut-off of proteins affected in 170 patients or less. Both cut-offs were defined based on the distribution. The colours of the proteins are from the representative network modules from Fig. 2 (HLA human leucocyte antigen).

We then investigated how the UC-associated signalling network was different in each of the 378 UC patients. Based on the set of SNPs present in each patient, we defined patient-specific UC-associated signalling networks, called ‘network footprints’. Unsupervised hierarchical clustering using different linkage algorithms of 378 patients stratified the patient-specific network footprints into four distinct clusters (Fig. 3a). The distribution of patients in the four clusters is presented in Supplementary Table 1.

SNP-affected proteins with many protein interactions drove the clustering of patients, often designated as hub proteins in network biology. In our analysis, we defined these proteins as ‘cluster driving proteins’ and the SNPs affecting them are identified in Table 1 (bold text). The SNP rs7404095 affecting *PRKCB* gene had the largest effect in clustering the patients, as it has 305 interactor partners in the network. PRKCB has been implicated in the pathogenesis of IBD due to its effects on the colonic mucosa^42^, colonic microbiota^43^ and cell junction complexes^44,45^. This SNP divides the patient cohort into two different clusters (Fig. 3a, b). The secondary divider for clusters is the SNP rs913678, which is in the enhancer region of *SNAI1*, *PTPN1* and *CEBPB*. SNAI1 is a transcription factor involved in epithelial-mesenchymal transition^46^. In dextran sulphate sodium (DSS)-induced colitis, it was shown that SNAI1 augmented the effects of MIST1 on the inflammasome protein NLRP3, promoting inflammation^47^. PTPN1 is a phosphatase that inhibits many tyrosine phosphate receptors such as EGFR^48^ or PDGFR^49,50^. Inhibiting PTPN1 increases angiogenesis and decreases inflammation^51^. CEBPB is a transcription factor overexpressed in both DSS- and beta caryophyllene-induced colitis^52^. Tertiary drivers are the SNP rs477515 affecting TFBSs in the enhancer region of *HLA* genes, and the SNP rs943072 affecting TFBS in a shared enhancer region of *VEGFA*, *XPO5* and *POLH*.

We used two additional network resources (Reactome^53^ and STRING^54^) to validate the clustering of the patients. From the 48 SNP-affected proteins 23 were present in Reactome and 33 in STRING. The UC-associated signalling networks were not similar, due to the complementarity of the three used networks (Supplementary Fig. 2). The clusters were driven by the primary hubs in the networks that were the various HLA proteins in Reactome and STRING, and the secondary drivers were the VEGFA, XPO5 and POLH proteins (Supplementary Data 4). These SNP-affected proteins divided the patient clusters tertiary and quaternary in the OmniPath network-based clustering (Supplementary results). The similarity of the patient clusters was low (adjusted rand index <0.05; Supplementary Fig. 1) but the Gene Ontology Biological Processes enriched in the networks were similar in all three networks, highlighting various immune functions (Supplementary Fig. 3, Supplementary Data 5).

Looking at the distribution of affected proteins in the patient cohort (Fig. 3c), we identified processes and proteins frequently affected in UC patients as well as more specific processes that were affected only in a smaller group of patients. In particular, we found that 63 proteins were affected in 79.5% of the patients (300 patients) that were involved in various immune system processes, autophagy and NFKB signalling (Supplementary Fig. 4, Supplementary Data 6). Also, 114 proteins were affected in less than 170 patients (Supplementary Fig. 4 and Supplementary Data 6) that were involved in cellular adhesion, angiogenesis and transmembrane receptor tyrosine kinase activity.

### Validating the iSNP clusters using an independent cohort

**Fig. 4:**
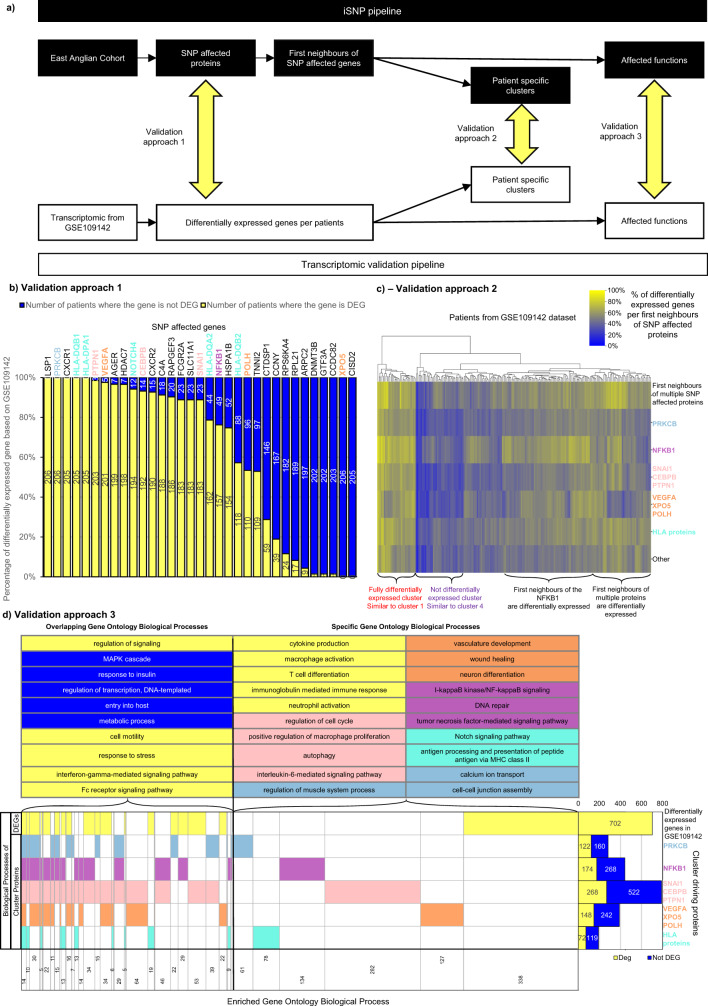
Validation of the iSNP method with transcriptomic data from an independent cohort of ulcerative colitis patients. **a** Flow chart depicting the validation approaches. **b** Single nucleotide polymorphism (SNP) affected genes differentially expressed in ulcerative colitis (UC) patients from biopsy samples of the GSE109142 dataset. Absolute log2 fold change > 1 was used as a cut-off. **c** The percentage of differentially expressed genes from the first neighbours of the cluster-driving proteins using the same dataset. Analysis of the patients in the independent cohort produced two clusters similar to those generated by iSNP (red and purple cluster on Fig. 3B). The most differentially expressed genes from the UC-associated signalling network in the validation cohort were the first neighbours of multiple SNP-affected proteins. **d** Similarities between the over-represented Gene Ontology Biological Processes between first neighbours of cluster-driving proteins and differentially expressed genes. Gene Ontology terms were considered enriched based on a Benjamini-Hochberg corrected hypergeometric test *p* < 0.05. A gene was considered differentially expressed based on |FC| > 1 and q < 0.05 Benjamini-Hochberg corrected moderate *t*-test There are two main groups of Gene Ontology Biological Processes: common and specific. The common processes include regulation of signalling or metabolic processes while the specific processes represent the cluster-driving protein and its cluster function or the transcriptomic effect of inflammation. Differentially expressed genes, first neighbours, or functions are represented in yellow and the cluster-driving genes are represented by their respective colours: pink—SNAI1, CEBPB, PTPN1; blue—PRKCB; orange—VEGFA, XPO5, POLH; purple—NFKB1, turquoise—HLA proteins.

To validate the iSNP methodology, we used the TAMMA resource^55^, which is the largest available transcriptomic resource in IBD where the origin of the patient biopsy is available. We identified the study GSE109142^39^ containing 206 juvenile, treatment-naive UC samples from their index colonoscopy (at diagnosis with active disease) and 20 juvenile controls. The data were coming from the PROTECT study^56^. We defined whether a gene is differentially expressed in the UC patients compared to controls using fold change as a simple metric and developed three validation approaches (Fig. 4a): (1) Using the SNP-affected genes to determine whether they are differentially expressed in the transcriptomic dataset; (2) Examining differentially expressed genes from the UC-associated signalling network in the transcriptomic dataset; (3) Comparing overlapping Gene Ontology Biological Processes of the SNP-affected proteins with the Gene Ontology Biological Processes of the differentially expressed genes from the transcriptomic dataset.

The first validation approach revealed that the SNP-affected genes were differentially expressed on average in 63.24% patients (SD = 39.58%) (Fig. 4b). Of the cluster-driving SNP-affected genes, *PRKCB* and two HLA genes, *HLA-DQB1* and *HLA-DPA1*, were differentially expressed in all patients in the validation cohort, whereas *VEGFA* and *CEBPB* were differentially expressed in 97.6% and 93.2% of the patients, respectively. This validation analysis demonstrated that the SNP-affected genes we have functionally annotated (predicted) were also differentially expressed in an independent cohort of UC patients.

The second approach (Fig. 4c) used the cluster-driving proteins and their first neighbour’s gene expression to compare the patient clusters generated from the transcriptomic measurements with the patient clusters generated from the iSNP pipeline. Two clusters were similar between the transcriptomic and the genomic datasets derived analyses. The first had all the SNP-affected genes and their first neighbours differentially expressed (the red cluster on Figs. 3b, 4c), with the second one containing only a few differentially expressed genes (the purple cluster on Figs. 3b, 4c). These clusters matched clusters 1 and 4 in the iSNP study analysis, respectively. The most differentially expressed genes in the analysis were genes that were first neighbours of more than one cluster-driving proteins, or the NFKB1-related first neighbours impacting the clustering of the transcriptome analysis. These results imply that the cluster-driving proteins highlighted by the iSNP workflow are also identified as being important in an independent cohort of UC patients. Moreover, we replicated the patient clustering with an independent cohort, and using only transcriptomic data with no genotype (SNP) data, further validating the power of the iSNP approach.

The third validation approach showed that the biological functions which we have identified using the UC-associated signalling network were also differentially regulated in the independent cohort. We identified the Gene Ontology biological processes that were overlapping between the differentially expressed genes and first neighbours of SNP-affected proteins (Fig. 4d, Supplementary Data 3). These included unspecific functions, such as metabolic process, regulation of signalling or cell motility. The overlapping biological processes which were not differentially expressed were upstream regulatory functions, such as MAPK cascade or response to insulin. Specific over-represented processes from the iSNP network analysis were upstream processes such as interleukin-6 mediated signalling, wound healing, and Notch signalling. The specific processes over-represented based on the differentially expressed genes from the validation cohort were downstream, inflammation-related processes including immune cell activation (e.g. T cell differentiation, neutrophil activation, macrophage activation). We also compared the over-represented gene ontology biological processes in the differentially expressed genes with those biological processes which were over-represented in the first neighbours of the cluster-driving proteins (side stacked bar chart in Fig. 4d). On average, 37.7% of the enriched biological processes were similar between the differentially expressed genes and the first neighbours of the cluster-driving SNPs.

Our validation approaches confirmed that the iSNP analysis identified the known genes involved in active UC. Moreover, with the increased coverage from the first neighbours of the SNP-affected proteins, iSNP enabled the identification of those genes and proteins that are involved in UC pathogenesis that would not have been identified by conventional genetic or transcriptomic analysis alone.

## Discussion

We have designed an integrated systems genomics workflow (Fig. 1, Supplementary Fig. 1), termed iSNP, to layer patient data from population-wide genomics with network biology and transcriptomics using UC as a model of a complex genetic disease. Our aim was to resolve the complex genetic background contributing to disease pathogenesis for an individual patient. To achieve this, we first identified so far hidden proteins involved in UC pathogenesis, second we identified key pathogenic pathways for UC and third we determined if patients had similar or different pathological processes in disease development. This was done with a view to providing insights that could advance personalised medicine for patients with UC. This study used functional annotation of non-coding SNPs with the integration of transcriptomics and protein–protein interactions at an individual patient level.

There are significant challenges in designing and executing computational pipelines for functional analysis of genetic data, particularly on an individual patient basis (see Supplementary Discussion for more detailed discussion). To overcome input challenges, we accessed high-quality individual patient genetic information from the UK IBD Genetic Consortium. This comprises preprocessed and quality-controlled immunochip data^57^, giving individual patient alleles present at SNP sites. This allowed us to functionally annotate UC-associated SNPs on a patient-by-patient basis. A binary approach was used for determining whether a SNP-affected the regulation of a gene or protein, allowing us to identify when a SNP weakly affects the binding of a transcription factor (TFBS) or miRNA target site (miRNA-TS), but does not eliminate the site completely, giving a broader overview of SNP functional annotation.

For functional annotation of SNPs within TFBS, we utilised the two widely cited, validated tools, Regulatory Sequence Analysis tools (RSAT)^58,59^ and Find Individual Motif Occurrences (FIMO)^60^. We considered the length of the TFBS query sequence to include promoters and enhancers. We acknowledge that not all TFBS in enhancer regions will be active, and that recently artificial intelligence techniques have integrated predictions of chromatin interaction with SNP data to identify SNPs in areas of active chromatin^10,11^. A switch mechanism to identify which TFBS were active or inactive was not available during the development or expansion of the iSNP workflow so we adopted a simple approach: If a TFBS was affected in an enhancer site by a SNP with a target gene in the Human Enhancer Disease Database (HEDD) it was retained within the network.

In terms of the miRNA-TS identification algorithm, both MIRANDA^61^ and TargetScan^62^ were trialled for inclusion in the pipeline. Both performed well; however, as TargetScan requires genome assembly to work, it was not plausible to integrate it into a functional annotation pipeline. Although SNPs may impact other parts of miRNA biogenesis and action, we utilised the site of SNP impact with the largest wealth of experimental data.

The UC-associated signalling network identified mechanisms of transcriptional and post-transcriptional regulation impacted by non-coding SNPs. There was more transcriptional regulation of SNP-affected genes than miRNA-based regulation (Fig. 2b) due to the significant number of SNPs annotating within TFBS in enhancer regions (Table 1). Each enhancer influences multiple genes and multiple transcription factors were predicted to bind to any given enhancer, meaning that each SNP had a pleiotropic but individually minor effect on the expression of various genes.

In contrast, the SNPs in miRNA-TSs have a specific effect on their individual target genes. Due to the fine-tuning role of miRNAs, the gain or loss of a miRNA-TS by itself has a small effect on the regulation of a cell^63^. iSNP mapped both the specific and pleiotropic regulatory changes one step further using a protein–protein interaction network. This has an inherent risk of increased noise within the network, and to reduce this we utilised the sparse OmniPath which integrates experimentally validated protein–protein interactions from 44 sources^40^. From studies of cancer-related signalling networks, we have shown that information regarding pathogenic pathways to disease can be gleaned from the direct protein–protein interactors for a protein of interest^33^. By integrating the protein–protein interaction and regulatory SNP effects, the iSNP method highlighted key pathogenesis pathways including calcium homeostasis, cell adhesion, stress response and cytokine signalling (Fig. 2a, b). We also compared the results we got using the OmniPath network with two other protein–protein interaction networks, and we found similar functions affected by SNPs. This confirmed that our findings did not depend on the specific network resource we used in the study.

The calcium homeostasis signalling pathway has not been identified previously as a driver of inflammation in UC. Intracellular calcium levels were described as altered in ulcerative colitis^64^ and described as a mechanism involved in DSS induced colitis in vitro^65^. However, closely linked with calcium homeostasis are Vitamin D signalling pathways, which have been hypothesised as a link between aberrant colonic mucosal vitamin D metabolism and the development of IBD^66,67^. Calcium homeostasis is likely linked to osteopenia and osteoporosis in IBD. Further investigation is required to decide what part of the intracellular or systematic calcium metabolism is affected in UC. There was not enough granularity in the clinical data, or a large enough population size, to determine if the cohorts of patients with affected calcium homeostasis had alterations in their bone mineral density compared to those patients without this pathway involvement, or to remove confounders such as recurrent corticosteroid therapy.

Pathways involved in the regulation and cellular response to stress, including wound healing and stress-related TFs, such as PPARs, were identified via NFKB1. Wound healing is complex and in the intestine involves multiple cell types, including immune cells, macrophages, fibroblasts, endothelial cells, intestinal epithelial cells and stem cells. Intracellularly, these pathways are also complex, but within the UC-associated signalling network, we identified the involvement of proteins integral to inflammasomes and peroxisomes. Specifically, within the UC-associated signalling network, we identified SNAI1, which is a regulator of the NLRP3 inflammasome^47^. There has been extensive analysis of the NLRP3 inflammasome and its role in IBD in both animal and in vitro studies, but the results are inconsistent, with the NLRP3 inflammasome being deleterious or protective depending on the colitis model used, the gut microbiota, or the means of inducing colitis in animal models^68^.

Pathways impacting immune cell motility and cellular adhesion in UC form the basis of therapeutic management with vedolizumab (a4b7 integrin inhibitor) and etrolizumab (b7 integrin subunit inhibitor). Neither gene was affected by a SNP within the network, nor in the first neighbours, but cell motility and adhesion pathways feature in a distinct subset of patients indicating a potential mechanism and explanation by which therapies that impact these pathways may be more or less successful in certain subsets of patients. This needs to be examined more closely and validated in a large clinical cohort, as it may be a means for personalising therapeutic strategies based on patient-specific underlying pathogenic mechanisms in UC.

From the individual patient networks, we undertook unsupervised clustering, which was driven by the highest degree nodes (hub) using distance metrics within a hierarchical agglomeration method. This allows us to identify structures within the networks, which were hitherto unknown. One limitation of this approach is a potential bias towards promiscuous hubs, which have high numbers of curated interactions within the interactome networks. An example of this is PRKCB. Conversely, these large hub proteins are very important to the network^69^ as they identify where a SNP has a wider effect on signalling pathways, and from this, we can identify particular pathways unique to clusters of patients which aim to correlate with therapeutic response or disease process. However, no significant differences based on the cohorts (Chi-square tests *p* > 0.05, One way ANOVA *p* > 0.05, Supplementary Table 1) were found. This is not unexpected as it required nearly 30,000 patients for Cleynen and colleagues to identify NOD2, MHC and 3p21 as being associated with the age of disease onset and disease location in IBD^70^.

Our analysis identified multiple genes whose translated proteins were hubs within the network including NFKB1 which is a central player in inflammatory signalling cascades, immune-mediated processes and in tight junctions regulation, but in our network was shown not to be a cluster-driving protein. The HLA proteins were cluster-driving proteins within the network, but did not include the known IBD HLA serotypes^71^ (HLA-DQB1 with Crohn’s^72^) with HLA-DQB2 and HLA-DPA1 being associations identified here. Unexpected cluster-driving proteins were identified that have clear links with IBD such as PRKCB, and VEGFA^73,74^ as well as proteins that have not been previously associated with UC including Exportin 5 and DNA polymerase eta. The involvement of Exportin 5 (a required protein for canonical miRNA biogenesis^75^), as well as the multitude of miRNA-TSs identified, adds weight to UC being a disease whose pathogenesis is intrinsically complex, with multiple small impacts on upstream gene regulation as opposed to singular high impact phenotypic mutations.

Whilst we have used UC as a use case study for iSNP, the pipeline is not disease-specific. We have made iSNP accessible and tailorable, accounting for the importance of functional annotation and downstream analysis of non-coding SNP effects for complex genetic diseases. iSNP is a dockerised pipeline that can be interfaced using the command line. Each of the analytical modules of the pipeline can be run independently of each other or run from start to finish. The parameters for each analytical module can be tuned by the user based on the input data. It is available on GitHub at https://github.com/korcsmarosgroup/iSNP.

The integrative SNP Network Platform (iSNP) is a workflow to functionally annotate non-coding SNPs, identify the first neighbour interactions within a disease-specific network and identify signalling pathways in which these SNPs and interactors are over-represented. iSNP has the functionality to allow this to be done on a broad scale to identify disease-associated pathways, and on an individual level to identify patient-specific affected pathways. Using UC as an example of a complex genetic disease, iSNP has identified how patients have differing mechanisms of pathogenesis. We identified pathways regulating the cellular response to stress, cell motility and calcium homeostasis as being over-represented in the UC-associated signalling network. Further work now needs to be done on larger cohorts and with multi-omics datasets to confirm the potential for iSNP to be used for precision therapy based on patient-specific genetics.

## Methods

### Sources of SNP data

UC-associated index SNPs were identified from the UK IBD Genetics Consortium Immunochip data^12^ and the Broad Institute Repository^76^. If no fine mapping was available for an index SNP (the immunochip finemapped SNP had an R^2^ < 0.8), then the highest proxy partners (based on tightest linkage disequilibrium and distance) were assessed using a SNP proxy search and were included in the analysis. Each SNP was annotated using Ensembl from the rsID using the genome map GRCH38.p7. Disease-associated SNPs were retrieved from the original data source.

After obtaining ethics approval from the University of East Anglia Faculty of Medicine and Health Science ethics committee (ref 02-01-16), anonymised individual patient immunochip data and clinical parameters for 378 patients were retrieved from the UK IBD Genetics Consortium from seven centres across East Anglia, UK (Cambridge, Norwich, Ipswich, Stevenage, Luton, Bedford and West-Suffolk). Informed consent of the patients was obtained by the IBD Bio-resource team. The patients have consented to the IBD Bio-resource consent form version 2. We included patients between 16 years and 83 years of age at diagnosis to account for the bimodal age prevalence of UC (See Supplementary Table 1 for patient demographics). SNPs were characterised into different types depending on their location in the genome: exonic (missense, synonymous), intronic/untranslated regions and intergenic. Flanking nucleotide sequences were obtained from the downloaded September 2017 version of dbSNP^77^. For the list of analysed SNPs and their effect, see Supplementary Data 1.

### Assessing the effect of SNPs on transcription factor binding sites and miRNA-TS

From the JASPAR database, 746 human transcription factors’ binding profiles represented by Position Specific Scoring Matrices (PSSMs) were downloaded^78^. The JASPAR format PSSMs were converted to the TRANSFAC format to ease handling of results. To assess the effect of the SNP on the gain or loss of putative TF binding sites, flanking sequences 50 bases upstream and downstream of the SNPs were extracted. The Regulatory Sequence Analysis Tool (RSAT) *matrix-scan*^58^ was used to search for potential TFBS in the ancestral and patient-specific mutant alleles. The background model estimation was determined by using residue probabilities from the genome version GRCH38.p7 sequences of all promoters based on the UCSC genome table browser^79^ 5KB before the TSS and all enhancers from the HEDD database^80^. In calculating the background probabilities we used a Markov order of 1. The search was subject to both strands of the sequences. Hits with a *P*-value ≤1e-05 were considered binding sites. Other parameters were set at default values.

As a complementary TF binding sites prediction algorithm, FIMO was used^60^. FIMO predicts the transcription factor targets sites using a matrix-based sequence scanning algorithm without a hidden Markov model, unlike the previous tool RSAT *matrix-scan*. It calculates the log-odds scores comparing random and test sequences followed by a Benjamini-Hochberg-based false discovery correction of the *P*-value. The false discovery rate cut-off was 0.1.

To increase the coverage of the TF binding sides, enhancer regions were added using the Human Enhancer Disease Database (HEDD)^81^. HEDD contains the enhancers from ENCODE^82^, FANTOM5^83,84^ and the Epigenomics RoadMap^85^. To assess the effect of the SNPs on miRNA-TSs, the 22 bp sequences of mature miRNAs were retrieved from miRBase^86,87^. The flanking sequences of SNPs were assessed for the presence of miRNA-TSs using miRanda^88^. Hits occurring in the seed region (2’–8’) of the miRNAs, and with alignment scores ≥90 and energy threshold ≤ −16 kcal/mol were considered as TS. Other parameters were set to default settings. TSs in the coding region or in the first intronic region were kept. A final manual check was performed to ensure that the SNPs overlapped with the predicted TFBS or miRNA target sites. For the miRNA-TS predictions, miRanda was chosen as it predicts and characterises miRNA binding sites using entropy-based binding energy scores instead of traditional conservation-based methods^88^. Gain or loss of the regulatory interactions between TFs and protein-coding genes were also considered where the protein-coding gene was in the promoter or in the enhancer region. We defined the promoter regions as 5 kb upstream from the transcription start site and downstream to the first exon of the gene. This information was retrieved using the feature retrieval function of the UCSC genome table browser^79^. The effect of SNPs on the uncovered TFBS or miRNA-TSs was classified into either a gain or loss of binding site/target site or a neutral change. Only those sites identified as loss or gain regarding sites corresponding to the ancestral allele were considered for subsequent analysis. We referred to genes corresponding to such SNPs as ‘SNP-affected genes’.

### Network construction and analysis

Protein–protein interactions of the proteins encoded by SNP-affected genes were obtained from OmniPath on 10 January 2020^40,41^. For the STRING network, we used stringent parameters using only the physical protein–protein interactions: values >0 in the experimental and database channel in the physical links downloaded on 28 October 2021^54^. For the Reactome interactions, we used the *Homo sapiens* mitab interaction file downloaded on 28 October 2021^89^. All interactions were translated to UniProt Accession numbers^90^ using the UniProt mapping tool with a python script. For each patient, the set of proteins encoded by SNP-affected genes and their first interactors (first neighbours) were defined as the UC-associated network footprint of a particular patient. The union of all network footprints, the UC-associated signalling network, was analysed and visualised in Cytoscape 3.3.0^91^ using the inverted self-organising map layout. We retained only those SNP-affected genes which were present in the OmniPath resource and which formed a giant component with their interactors. Patient-specific networks were constructed using the Cytoscape CyRestClient 0.6 in Python 3.6^92^.

Module analysis was carried out using the Clustermaker2 1.1.0 Cytoscape app^93^ implementing the GLay clustering method^94^, which is an implementation of the Girvan-Newman clustering algorithm^95^. Briefly, the clustering method deletes the highest betweenness edges from the network until the network collapses to non-connected components and these components form the clusters. We used this clustering method due to being algorithmically quick and giving biologically meaningful clusters. (For further discussion see Supplementary Discussion). We call the network clusters ‘modules’, to distinguish them from patient clusters.

### Hierarchical clustering and statistical analysis

The *scikit-learn (v 0.23.)* package was used for hierarchical clustering of the patient-specific clusters^96^. The constructed distance matrix between patients was based on the Hamming distance^97^. If a protein was directly or indirectly affected by a SNP, it was assigned a value of “1” for a patient. If the protein was not affected, it was scored as “0”. The cluster similarity was measured using the adjusted rand index from the python Scikit-learn package^96^.

### Gene Ontology analysis

The Gene Ontology analysis was performed using the GORILLA tool^98^. The gene ontology biological processes were visualised using REVIGO^99^. For the overrepresentation test, the background was the giant component of the specific network resource (OmniPath, Reactome, or STRING). The tests were false discoveries corrected by the Benjamini-Hochberg method. We considered a Gene Ontology Biological Process term representative for a cluster if it was enriched with a corrected q < 0.05.

### Validation cohort analysis

The TAMMA transcriptomics collection datasets were downloaded on 14 June 2021^55^. After examining the metadata, the study GSE109142^39^ was used as it had annotated source tissue and an adequate number of patients and controls (206 and 20, respectively). Expression tables were assembled from the gene-specific expression values remaining those genes expressed in 10 or more read counts and the samples were normalised using the limma package (version 3.50.1)^100^ which implemented voom^101^. The log2 normed counts were used for further analysis. On a patient to patient basis, the fold change values were calculated by comparison with the average of the control samples. If the absolute differential expression was >1 then the gene was considered to be differentially expressed in that patient. This binary matrix was used for clustering and visualisation.

For case one, only the SNP-affected genes in the OmniPath database were used (Table 1). For case two, the UC-associated signalling network was used with the proteins grouped by the hub SNPs. For case three, differentially expressed genes in GSE109142 were used to compare the SNP-affected genes’ first neighbours enriched gene ontology biological processes. The definition of differentially expressed genes was |FC| > 1 and q < 0.05 Benjamini-Hochberg corrected moderate *t*-test using the standard limma analysis pipeline^100^.

### Reporting summary

Further information on research design is available in the Nature Research Reporting Summary linked to this article.

## Supplementary information

Supplementary Information

Reporting Summary

Peer Review File

Description of Additional Supplementary Files

Supplementary Data 1

Supplementary Data 2

Supplementary Data 3

Supplementary Data 4

Supplementary Data 5

Supplementary Data 6

Supplementary Data 7

## Data Availability

The immunochip SNP data were retrieved from the IBD bioresource database https://www.ibdbioresource.nihr.ac.uk/. The data are available under restricted access due to the clinical and so sensitive nature of the data. Access can be obtained by applying to the IBD Bio-resource through https://www.ibdbioresource.nihr.ac.uk/index.php/resources/applying-for-access-to-the-ibd-bioresource-panel-2/. The outcome of the pipeline is available in Supplementary Data 7 containing internal patient IDs, SNP-affected genes and the transcription factors and miRNAs. The transcriptomic data were downloaded from the GEO database accession: GSE109142.

## Code availability

The iSNP pipeline is available in the project GitHub page: https://github.com/korcsmarosgroup/iSNP, 10.5281/zenodo.6346651.
