# Supplementary material for: A systems genomics approach to uncover patient-specific pathogenic pathways and proteins in ulcerative colitis
Source: Nat Commun. 2022 Apr 28;13:2299. doi: 10.1038/s41467-022-29998-8 (PMC9051123; doi:10.1038/s41467-022-29998-8)
Supplement: Supplementary file 1 — Supplementary Information [file 41467_2022_29998_MOESM1_ESM.pdf]

A systems genomics approach to uncover patient-specific pathogenic pathways and proteins in ulcerative colitis

Johanne Brooks-Warburton, Dezso Modos, Padhmanand Sudhakar, Matthew Madgwick, John P. Thomas, Balazs Bohar, David Fazekas, Azedine Zoufir, Orsolya Kapuy, Mate Szalay-Beko, Bram Verstockt, Lindsay J Hall, Alastair Watson, Mark Tremelling, Miles Parkes, Severine Vermeire, Andreas Bender, Simon R. Carding, Tamas Korcsmaros

Supplementary Information

Supplementary Results

The STRING based UC-associated signalling network contained 709 proteins and 11016 protein-protein interactions, meanwhile the Reactome based UC-associated signalling network contained 317 proteins and 1867. Out of the 48 SNP affected proteins we found in our study cohort, 33 and 23 were in the STRING and Reactome derived networks respectively. The patient specific networks were clustered with the same clustering method that was used in the iSNP method (see Methods). The STRING derived network clustered the patients into 6 clusters, meanwhile using the Reactome derived network clustered the patients into 4 clusters (Figure S2 a and b). The Reactome and STRING clustering were dissimilar compared to the OmniPath derived network clustering (Figure S2 c and d). The STRING and the Reactome derived clusters were similar, only the STRING clusters were splitting two Reactome clusters into two (Figure S2e). We measured the similarity using the rand index (Figure S2 f). The primary cluster driving proteins in both the STRING and Reactome derived networks were affected by the SNP rs477515, which is related to various HLA proteins, NOTCH4 and AGER (Table 1). The secondary cluster driving proteins were affected by the SNP rs913678. These proteins included VEGFA, XPO5 and POLH. The clustering difference between the networks originated from the degree of these proteins. In Reactome and STRING, the HLA proteins were the highest degree proteins in the UC associated signalling network, meanwhile, in the OmniPath network, these proteins had not had as many interactions as PRKCB, NFKB1 or CEBPB. Important however to note that the affected biological processes in the UC-associated signalling networks were independent of the network source, indicating that the curation or other resource related biases do not affect our main message on the cellular functions affected in UC.

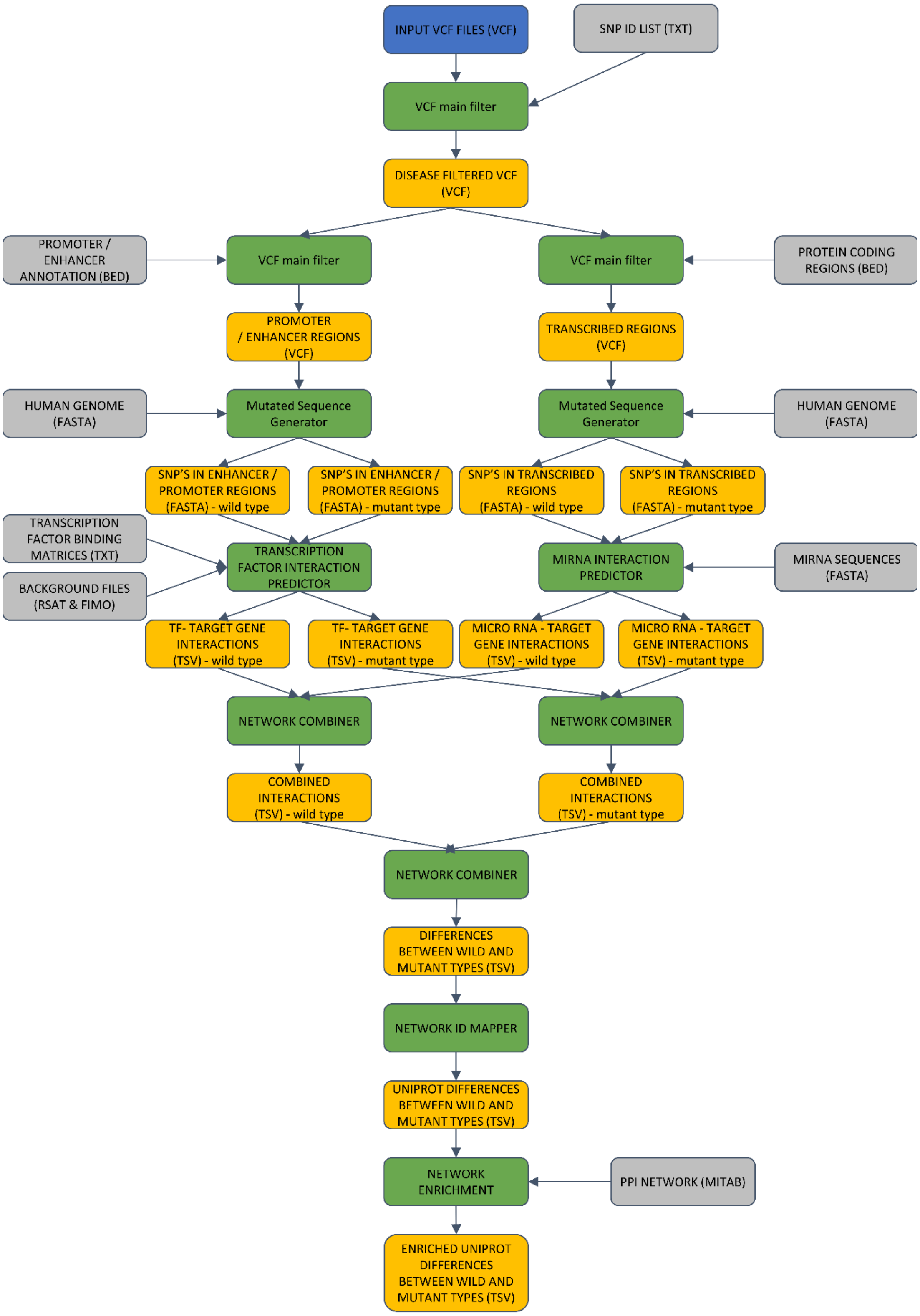

Supplementary Figure 1. The iSNP pipeline blue: input data, green: pipeline script, grey: external information, orange: output The iSNP pipeline uses the vcf files and various external information to find the SNPs location and search for the transcription factor binding sites or miRNA target sites for both the SNP affected – mutated - and not affected - wild type - sequence. After that the mutated and wild type network is compared and where are differences those differences are kept.

This analysis suggests that the OmniPath derived network has overlapping biological processes with the other two databases. OmniPath's coverage is higher compared to the two other databases: 34 compared to 33 and 23 (Figures S3 a). The low overlap at the node and the edge level shows that all three networks contain complementary information (Figure S3 b,c). The patient clustering can be database-specific, and would be driven by the degree of the SNP affected proteins - in our case the HLA proteins in the Reactome and the STRING derived networks and PRKCB in the OmniPath derived network. The enriched biological processes were overlapping between the network sources but the OmniPath network has a much higher number of enriched biological processes (Figure S3 d,e,f). All three networks contained the same immune related functions such as "Immune Related Process".

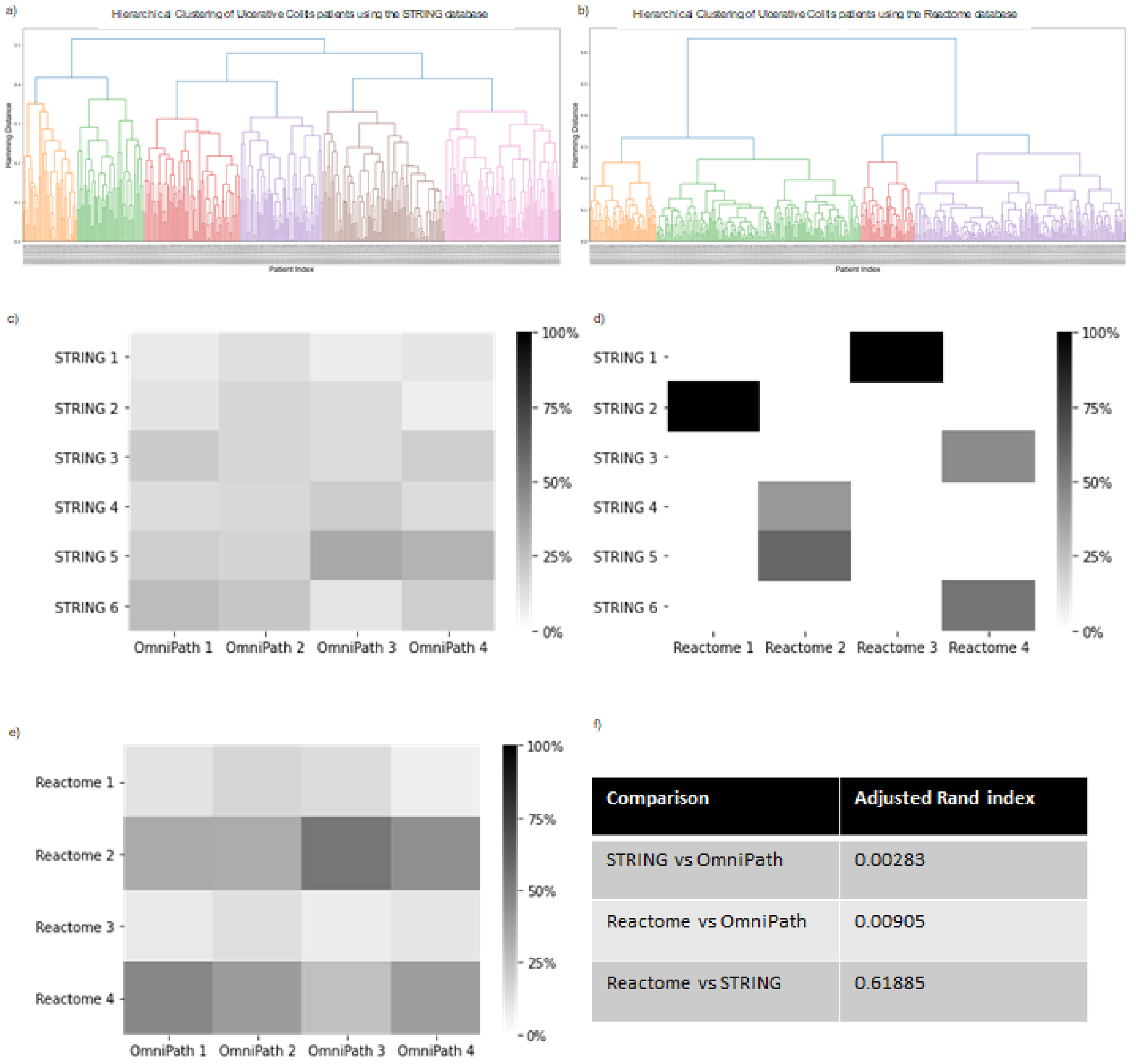

Comparison	Adjusted Rand index
STRING vs OmniPath	0.00283
Reactome vs OmniPath	0.00905
Reactome vs STRING	0.61885

Supplementary Figure 2. Running the iSNP method on two different networks. a) Hierarchical clustering of patients using the STRING network, Hamming distance. The patients are separated into six distinguishable clusters. b) Hierarchical clustering of patients using the Reactome network, Hamming distance. The patients are separated into four distinguishable clusters. c-e Heatmaps showing the similarity between clustering. Each cell in the heatmap represents the percentage of the patients in that column. c) Overlaps between the OmniPath and STRING clusters. d) Overlaps between

the OmniPath and Reactome clusters. In these comparisons, the percentages are in the OmniPath clusters. The clusters are not mapped to one another, meaning the databases measure different parts of the human interactome. e) Comparison between Reactome and STRING The clustering is similar; only the Reactome 2 and 4 clusters are the STRING 4,5 and 3,6 clusters, respectively. Here the percentages are in the Reactome clusters. f) Adjusted rand index calculations.

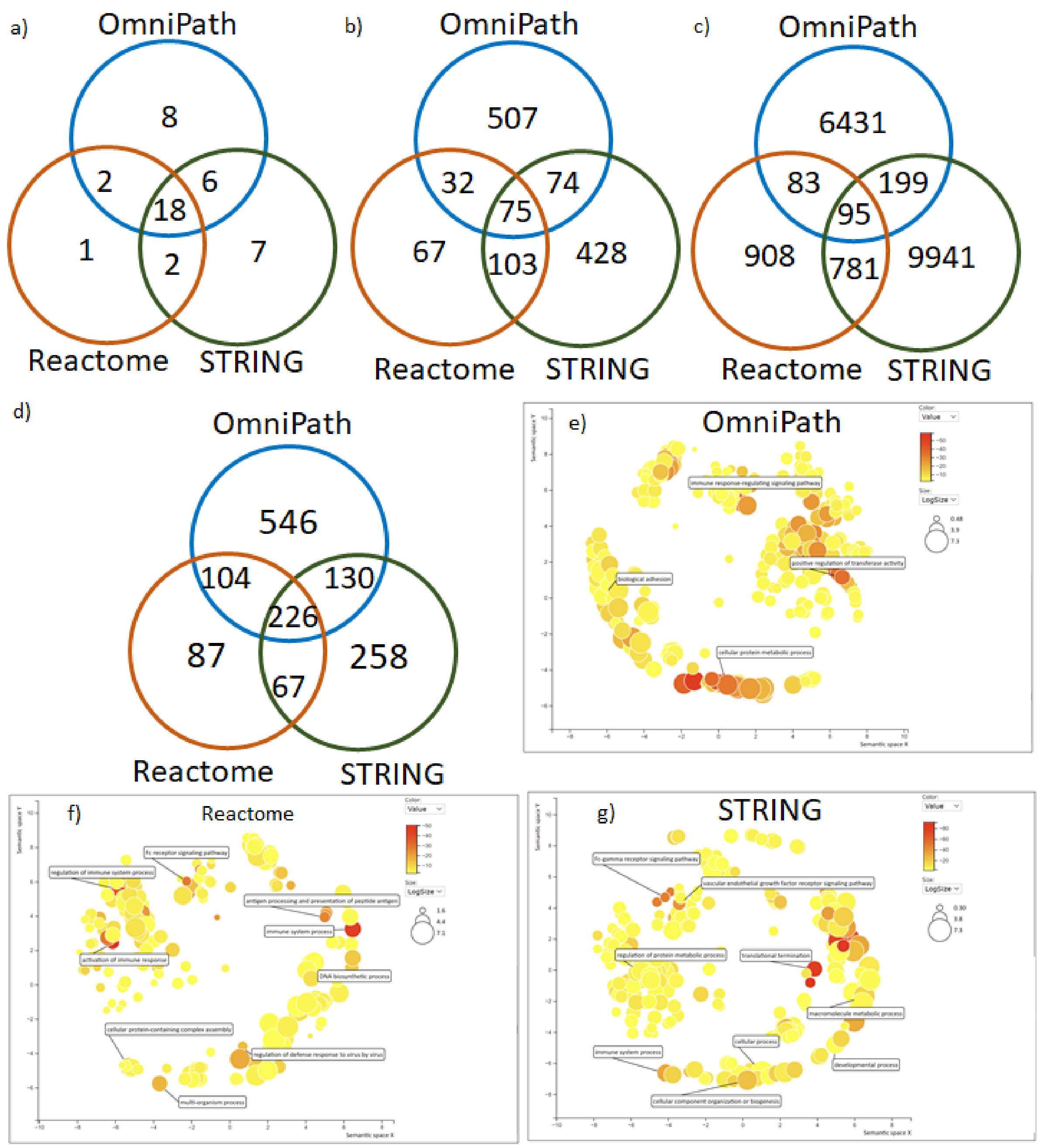

Supplementary Figure 3. Overlaps between various UC associated signalling networks using different initial query human interactome. a) SNP-affected proteins, b) Proteins (nodes), c) Physical protein-protein interactions (edges) d) Gene Ontology Biological Processes (functions) e) Enriched GO biological processes in the OmniPath network visualised by Revigo, f) Enriched GO biological processes in the Reactome network visualised by Revigo, g) Enriched GO biological processes in the OmniPath network visualised by Revigo The networks have high overlap at the initial SNP affected proteins, but they contain different edges and through that different interactors. However, the affected functions are similar. Benjamini-Hochberg corrected hypergeometric tests q<0.05 visualised.

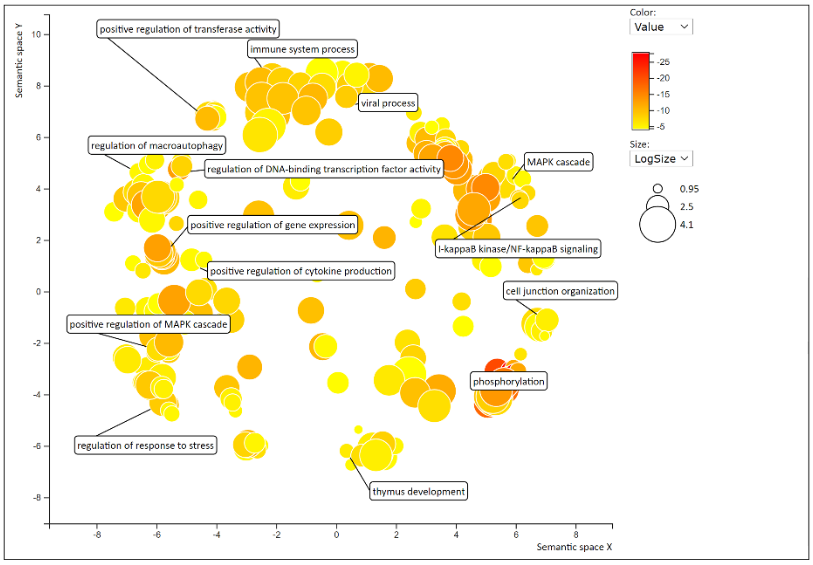

Biological process in proteins affected directly or indirectly in more then 300 patients

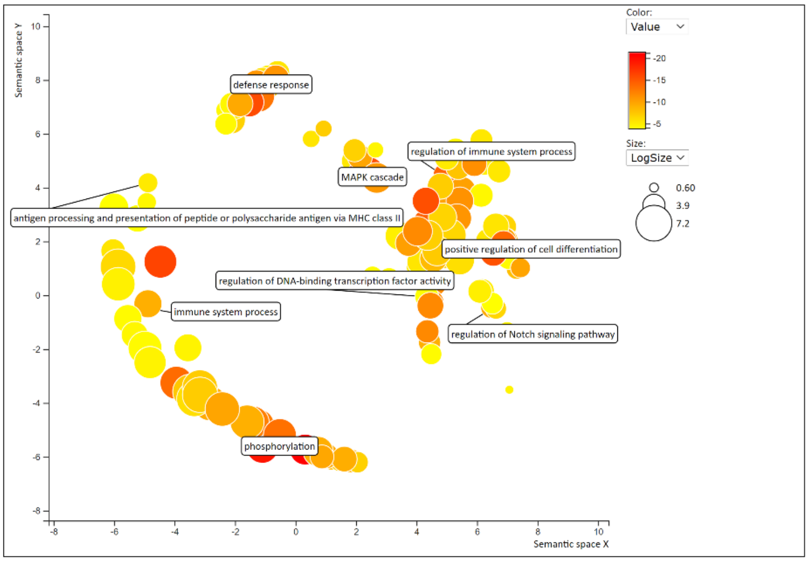

Biological process in proteins affected directly or indirectly in more then 170 but less then 300 patients

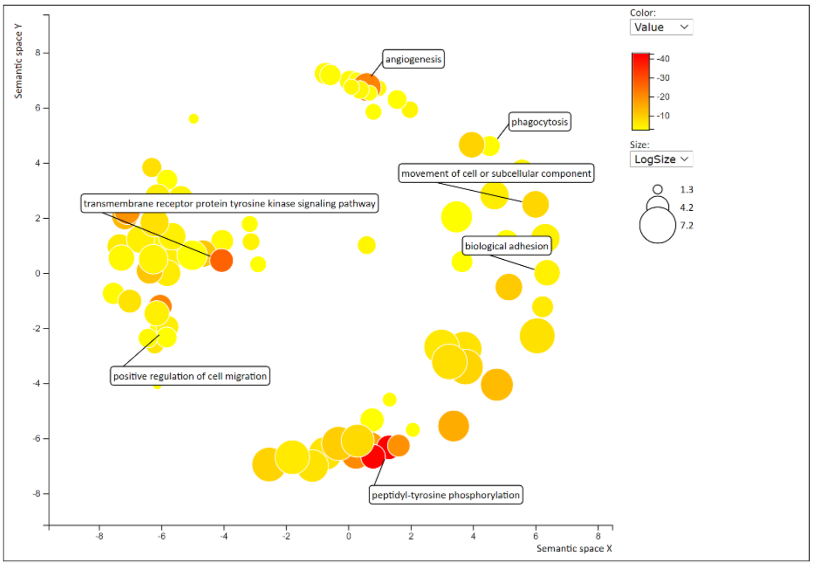

Biological process in proteins affected directly or indirectly in less then 170 patients

Supplementary Figure 4. Revigo analysis of overrepresented Gene Ontology Biological processes in commonly affected proteins grouped by in how many patients they are affected. A) Common biological process - affected in more than 300 patients. The processes involved in most ulcerative colitis patients

in our cohort are well known pathogenetic avenues of ulcerative colitis such as NFKB signalling and regulation of autophagy. B) Biological processes of genes affected between 300 and 170 patients. Note Notch signalling and MHC class II antigen presentation. These are functions of SNP affected proteins which are not involved at the first few clustering level. C) Specific processes – affected in less than 170 patients. Note angiogenesis and cell migration, which are probably related to the first neighbours of VEGFA. Benjamini-Hochberg corrected hypergeometric tests, q<0.05 visualised.

Supplementary Table 1 List of clustered patients and patient demographics

Cluster	Gender		Immunomodulator			Age		Age at diagnosis	
	Female	Male	No only ASA	Yes	No data	Mean	STD	Mean	STD
1	70	75	96	47	2	63	15.3	39.90648	15.35
2	50	61	74	36	1	60.1	13	36.72477	13.23
3	34	31	41	21	3	56.6	14.8	36.08065	15.61
4	28	29	35	22	0	57.9	15.4	36.4	14.32
Whole data set	182	196	246	126	6	60.3	14.7	37.78	14.67

STD: standard deviation, ASA: aminosalicylic acid

Supplementary Discussion

The conclusion drawn from computational workflows is dependent on three factors; 1) the quality of the input data, 2) the quality of the processing and parameter optimisation and 3) the quality of the analysis [1]. If any one of these components fails, then the entire pipeline fails leading to aberrant conclusions. We address each of these areas below.

Challenges with the input data

The high-quality individual genetic information was collected from the UK IBD Genetic Consortium. The data were preprocessed and quality controlled with immunochip data, giving the individual patient alleles present at the SNP sites. Immunochip contains a subset of specific SNPs associated with autoimmune diseases [2]. We looked for individual patient SNP burdens against IBD SNPs that had been finemapped. In this way, we were not analysing the data looking for novel SNPs. Our aim was to functionally annotate those that had been already identified on a patient by patient basis. The East Anglian Cohort was chosen due to the access to granularity of clinical data, to assess if clinical parameters could be associated with the patient stratification. This was ambitious, given that it required nearly 30,000 patients for Cleynen et al to identify NOD2, MHC and 3p21 as being associated with age of disease onset and disease location [3].

For validation ideally we should use a high number of patients with transcriptomic and genomic features, however that was not yet available. Instead, we turned to a validation cohort with transcriptomics data on a high number of similar patients from the PROTECT study [4]. The PROTECT cohort however has its own limitations [4,5]. It has only juvenile ulcerative colitis patients and a relatively low number of controls (n=20). However using a homogenous and treatment naive population we were able to demonstrate the similarities between the effect of the SNPs and the transcriptomic manifestation of UC.

During the testing phase of the pipeline design, it became clear that generating patient specific backgrounds for RSAT resulted in differing SNP effects of the same SNP. This was optimised by using a singular genetic background file for RSAT and FIMO to remove any differences between the patient data except for the SNP alleles, thereby reducing false positive/negative prediction. Whilst this allowed us to meet our aim of specific analysis of the SNP function, we acknowledge that it necessitates simplification of the complexity of the individual human genome.

We used a binary approach as to whether a SNP affects the regulation of a gene or protein - this allows us to identify when a SNP weakly affects the binding of a transcription factor or miRNA target site, but does not eliminate the site completely, giving a broader overview of SNP functional annotation.

One of the final input challenges we had to overcome was that the immunochip data we had to use was based on a 15 years old genome version, Hg18. We had to map the genomic coordinates to the better annotated Hg19 genome version. Such lift-overs are inherent with difficulties [6]; reassuringly we did not have any variants that would not map over due to it being in a contig from the Hg18 that did not reside in Hg19, or merged rs numbers. Our main issue was some variant alleles being recognised as ancestral alleles in Hg19. Each of these SNPs needed to be separated for manual filtering and checking.

Challenges with data processing and parameter optimisation

Transcription factor binding sites:

There are multiple transcription factor binding site prediction methods including hidden Markov models, hierarchical mixture models, support vector machines, and discriminative maximum conditional likelihood which all rely on prior knowledge such as position weight matrix. We utilised the two widely cited, validated tools - Regulatory Sequence Analysis tools (RSAT) [7] [8] and Find Individual Motif Occurrences (FIMO) [9].

There are known complexities with SNPs affecting transcription factor binding sites, including trans effects of SNPs. During optimisation, we took into account that the length of the transcription factor binding site query sequence needs to be the length of the binding site plus the SNP nucleotide, but this does not take into account the folding of DNA and trans effects of SNPs, or SNPs in promoters or enhancers so we utilised a longer nucleotide sequence to encompass this. We extensively analysed enhancer databases, of which there are many. [10–12], and utilised the largest repositories available. We acknowledge that not all transcription factor binding site enhancers will be active, and that very recently artificial intelligence techniques have been utilised to integrate predictions of chromatin interaction with SNP data to identify SNPs in areas of active chromatin [13,14], however a switch mechanism to identify which are active or not, was not available during the development and expansion of iSNP, so we used a simple approach: if a transcription factor binding site was affected in an enhancer site by a SNP with a target gene in the HEDD database, we kept it within the network.

For promoter regions, we undertook significant parameter optimisation. The complexity lies within the definition of a promoter which varies within the literature. Initially we used 2kb from the start of the genes in both directions looking for UC SNPs but this missed a significant number of ‘distant’ promoters, so we expanded the analysis to 5kb. Whilst this does not allow analysis of the multiple transcription start sites, it does give a global overview of SNP effects on individual transcription factor binding sites, so this pay off met our aim.

MiRNA Target Sites:

MiRNA target sites were identified within untranslated regions of the transcripts and first introns. To remove false positive/false negative results, any hits outside of these regions were excluded. In terms of the identification algorithm, we trialled both MIRANDA [15] and TargetScan [16] for inclusion into the pipeline - both of which perform well in miRNA target site identification, MIRANDA uses an algorithm which changes score dependent on the input nucleotides, whereas the TargetScan requires genome assembly to work, which make it a useful standalone, but as part of an functional annotation pipeline it was not plausible to integrate it. We acknowledge that SNPs may impact on other parts of miRNA biogenesis and action, however we utilised the site of SNP impact with the largest wealth of experimental data.

Challenges with the data analysis

The next challenge was how to identify key protein drivers of disease from within the networks. We have previously identified from studies of cancer networks, information regarding pathogenic pathways to disease can be gleaned from the direct protein-protein interactors with the protein of interest [17]. In our current use case, we identified the direct interactors of any SNP affected gene product (protein). This was via a transcription factor binding site enhancer, or promoter, or a transcript with an affected miRNA target site. Whilst there are datasets for gene splicing sites and pre-miRNAs, that would add a level of complexity, they have an increased risk of significant false positivity and negativity, so for this use case, the decision was made that these datasets need further validation before adding into the workflow.

Regarding utilising the East Anglian Cohort: Despite having good coverage of patient metadata from the UK IBD Genetic Consortium, supervised clustering did not identify any association with clinical parameters. Areas with gaps in the UK Genetic Consortium patient metadata included 2% of the treatment data and only 43% of the extent of the disease. We are therefore, able to say with confidence, that there is no generalisable correlation between the clusters and age of diagnosis, or requirement of treatment escalation. Given the gaps in the metadata, we are unable to analyse the correlation between disease extent or the

requirement of surgery. To answer these questions; larger cohorts, with breadth and depth of clinical data, is required, such as provided by Gut Reactions.

For network modularisation we used the top down Grivan-Newman clustering which was implemented in Cytoscape's Clustermaker app[18]. All network modularisation algorithms give back slightly different network modules due to the modularity plateau (equally good module determiantions)[19]. However the enriched functions in each module depend on the SNP-affected proteins and their network topology. We have not analysed the various structures of the network modules but tested how affected functions change using different networks (OmniPath[20], STRING[21], and Reactome[22] (Supplementary Figure S3 d-g, and Supplementary Table 6). We found that the enriched functions were similar if we used different network resources.

Supplementary References

1. Shade, A. & Teal, T. K. Computing workflows for biologists: A roadmap. *PLoS Biol.* **13**, e1002303 (2015).
2. Márquez, A. *et al.* Meta-analysis of Immunochip data of four autoimmune diseases reveals novel single-disease and cross-phenotype associations. *Genome Med.* **10**, 97 (2018).
3. Cleynen, I. *et al.* Inherited determinants of Crohn's disease and ulcerative colitis phenotypes: a genetic association study. *Lancet* **387**, 156–167 (2016).
4. Hyams, J. S. *et al.* Factors associated with early outcomes following standardised therapy in children with ulcerative colitis (PROTECT): a multicentre inception cohort study. *Lancet Gastroenterol. Hepatol.* **2**, 855–868 (2017).
5. Haberman, Y. *et al.* Ulcerative colitis mucosal transcriptomes reveal mitochondriopathy and personalized mechanisms underlying disease severity and treatment response. *Nat. Commun.* **10**, 38 (2019).
6. Pan, B. *et al.* Similarities and differences between variants called with human reference genome HG19 or HG38. *BMC Bioinformatics* **20**, 101 (2019).
7. Turatsinze, J.-V., Thomas-Chollier, M., Defrance, M. & van Helden, J. Using RSAT to

scan genome sequences for transcription factor binding sites and cis-regulatory modules. *Nat. Protoc.* **3**, 1578–1588 (2008).

8. Medina-Rivera, A. *et al.* RSAT 2015: regulatory sequence analysis tools. *Nucleic Acids Res.* **43**, W50-6 (2015).
9. Grant, C. E., Bailey, T. L. & Noble, W. S. FIMO: scanning for occurrences of a given motif. *Bioinformatics* **27**, 1017–1018 (2011).
10. Chen, H. & Liang, H. A High-Resolution Map of Human Enhancer RNA Loci Characterizes Super-enhancer Activities in Cancer. *Cancer Cell* **38**, 701-715.e5 (2020).
11. Sun, H. *et al.* ETph: enhancers and their targets in pig and human database. *Anim. Genet.* **51**, 311–313 (2020).
12. Wang, Z. *et al.* HEDD: human enhancer disease database. *Nucleic Acids Res.* **46**, D113–D120 (2018).
13. Meng, X.-H., Xiao, H.-M. & Deng, H.-W. Combining artificial intelligence: deep learning with Hi-C data to predict the functional effects of non-coding variants. *Bioinformatics* **37**, 1339–1344 (2021).
14. Xu, C. *et al.* Quantifying functional impact of non-coding variants with multi-task Bayesian neural network. *Bioinformatics* **36**, 1397–1404 (2020).
15. Betel, D., Wilson, M., Gabow, A., Marks, D. S. & Sander, C. The microRNA.org resource: targets and expression. *Nucleic Acids Res.* **36**, D149-53 (2008).
16. Agarwal, V., Bell, G. W., Nam, J.-W. & Bartel, D. P. Predicting effective microRNA target sites in mammalian mRNAs. *eLife* **4**, (2015).
17. Módos, D. *et al.* Neighbours of cancer-related proteins have key influence on pathogenesis and could increase the drug target space for anticancer therapies. *NPJ Syst. Biol. Appl.* **3**, 2 (2017).
18. Morris, J. H. *et al.* clusterMaker: a multi-algorithm clustering plugin for Cytoscape. *BMC Bioinformatics* **12**, 436 (2011).
19. Good, B. H., de Montjoye, Y.-A. & Clauset, A. Performance of modularity maximization in practical contexts. *Phys. Rev. E Stat. Nonlin. Soft Matter Phys.* **81**, 046106 (2010).

20. Türei, D. *et al.* Integrated intra- and intercellular signaling knowledge for multicellular omics analysis. *Mol. Syst. Biol.* **17**, e9923 (2021).

21. Szklarczyk, D. *et al.* The STRING database in 2021: customizable protein-protein networks, and functional characterization of user-uploaded gene/measurement sets. *Nucleic Acids Res.* **49**, D605–D612 (2021).

22. Jassal, B. *et al.* The Reactome Pathway Knowledgebase. *Nucleic Acids Res.* **48**, D498–D503 (2020).
