# Supplementary material for: A systems genomics approach to uncover patient-specific pathogenic pathways and proteins in ulcerative colitis
Source: Nat Commun. 2022 Apr 28;13:2299. doi: 10.1038/s41467-022-29998-8 (PMC9051123; doi:10.1038/s41467-022-29998-8)
Supplement: Supplementary file 2 — Reporting Summary [file 41467_2022_29998_MOESM2_ESM.pdf]

nature portfolio

Corresponding author(s): Tamas Korcsmaros

Last updated by author(s): Mar 28, 2022

Reporting Summary

Nature Portfolio wishes to improve the reproducibility of the work that we publish. This form provides structure for consistency and transparency in reporting. For further information on Nature Portfolio policies, see our Editorial Policies and the Editorial Policy Checklist.

Statistics

For all statistical analyses, confirm that the following items are present in the figure legend, table legend, main text, or Methods section.

n/a	Confirmed	
☐	☒	The exact sample size (*n*) for each experimental group/condition, given as a discrete number and unit of measurement
☐	☒	A statement on whether measurements were taken from distinct samples or whether the same sample was measured repeatedly
☐	☒	The statistical test(s) used AND whether they are one- or two-sided<br>*Only common tests should be described solely by name; describe more complex techniques in the Methods section.*
☐	☒	A description of all covariates tested
☐	☒	A description of any assumptions or corrections, such as tests of normality and adjustment for multiple comparisons
☐	☒	A full description of the statistical parameters including central tendency (e.g. means) or other basic estimates (e.g. regression coefficient) AND variation (e.g. standard deviation) or associated estimates of uncertainty (e.g. confidence intervals)
☐	☒	For null hypothesis testing, the test statistic (e.g. *F*, *t*, *r*) with confidence intervals, effect sizes, degrees of freedom and *P* value noted<br>*Give P values as exact values whenever suitable.*
☐	☒	For Bayesian analysis, information on the choice of priors and Markov chain Monte Carlo settings
☐	☒	For hierarchical and complex designs, identification of the appropriate level for tests and full reporting of outcomes
☒	☐	Estimates of effect sizes (e.g. Cohen's *d*, Pearson's *r*), indicating how they were calculated

Our web collection on statistics for biologists contains articles on many of the points above.

Software and code

Policy information about availability of computer code

Data collection	https://github.com/korcsmarosgroup/iSNP<br>Data was collected: IBD bioresource database<br>The control data was downloaded from the Gene Expression Omnibus: GSE109142
Data analysis	https://github.com/korcsmarosgroup/iSNP<br>Cytoscape version 3.3.0 with Clustermaker2 1.1.0<br>Validation: R 4.0.3 with limma 3.50.1

For manuscripts utilizing custom algorithms or software that are central to the research but not yet described in published literature, software must be made available to editors and reviewers. We strongly encourage code deposition in a community repository (e.g. GitHub). See the Nature Portfolio guidelines for submitting code & software for further information.

Data

Policy information about availability of data

All manuscripts must include a data availability statement. This statement should provide the following information, where applicable:

- Accession codes, unique identifiers, or web links for publicly available datasets
- A description of any restrictions on data availability
- For clinical datasets or third party data, please ensure that the statement adheres to our policy

The iSNP pipeline is available in GitHub. https://github.com/korcsmarosgroup/iSNP, DOI:10.5281/zenodo.6346651
The immunochips SNP data was retrieved from the IBD bioresource database https://www.ibdbioresource.nihr.ac.uk/. The data are available under restricted access due to the clinical and so sensitive nature of the data. Access can be obtained by applying to the IBD Bioresource through https://

www.ibdbioresource.nihr.ac.uk/index.php/resources/applying-for-access-to-the-ibd-bioresource-panel-2/.
The outcome of the pipline is available in Supplementary Data 8 containing patient IDs, SNP affected genes and the trtanscripton factors and miRNAs.
The transcriptomic data was downloaded from the GEO database accession: GSE109142.

Field-specific reporting

Please select the one below that is the best fit for your research. If you are not sure, read the appropriate sections before making your selection.

☒ Life sciences ☐ Behavioural & social sciences ☐ Ecological, evolutionary & environmental sciences

For a reference copy of the document with all sections, see nature.com/documents/nr-reporting-summary-flat.pdf

Life sciences study design

All studies must disclose on these points even when the disclosure is negative.

Sample size	378 patients from East Anglia ulcerative colitis cohort. We took all available patients for analysis. The data analysis was hypothesis free in the sense that we wanted to see the difference pathogenesis of these patients.
Data exclusions	No
Replication	Each patient had one particular immunochip, which was stored in the bio resource. Our study is a bioinformatical workflow. We have checked the parameters by multiple quality control replicates, but the end replication is n=1 of each patient.
Randomization	The cohort was not randomized. the patients were used as it is.
Blinding	We were not compared two variables when blinding is necessitated. It was a patophisiology study

Reporting for specific materials, systems and methods

We require information from authors about some types of materials, experimental systems and methods used in many studies. Here, indicate whether each material, system or method listed is relevant to your study. If you are not sure if a list item applies to your research, read the appropriate section before selecting a response.

Materials & experimental systems

n/a	Involved in the study	
☒	☐	Antibodies
☒	☐	Eukaryotic cell lines
☒	☐	Palaeontology and archaeology
☒	☐	Animals and other organisms
☐	☒	Human research participants
☒	☐	Clinical data
☒	☐	Dual use research of concern

Methods

n/a	Involved in the study	
☒	☐	ChIP-seq
☒	☐	Flow cytometry
☒	☐	MRI-based neuroimaging

Human research participants

Policy information about studies involving human research participants

Population characteristics	Patients of between 16 years and 83 years of age at diagnosis to account for the bimodal age prevalence of UC. 182 Female 196 Male 246 getting only mesalazine therapy 126 getting other therapeutic for 6 patients therapeutic information was not available. The age mean age the study recruitment was 60.3 (+/- 14.7 SD) years and the age of diagnosis mean was 37.8 years (+/- 14.7 SD).
Recruitment	We used the UK East Anglia cohort of 378 patients from the UK IBD genetics consortium for this analysis. The patients were recruited from seven centres across East Anglia, UK (Cambridge, Norwich, Ipswich, Stevenage, Luton, Bedford, and West-Suffolk)
Ethics oversight	Informed consent was obtained for the IBD Bio-resource. This research was done via the IBD Bio-resource data. The patients were consented by the bio-resource consent form version 2. The local ethics is to use the already consent patients for further study. University of East Anglia Faculty of Medicine and Health Science Ethics Committee REF: 02-01-16

Note that full information on the approval of the study protocol must also be provided in the manuscript.
