# Supplementary material for: A systems genomics approach to uncover patient-specific pathogenic pathways and proteins in ulcerative colitis
Source: Nat Commun. 2022 Apr 28;13:2299. doi: 10.1038/s41467-022-29998-8 (PMC9051123; doi:10.1038/s41467-022-29998-8)
Supplement: Supplementary file 4 — Description of Additional Supplementary Files [file 41467_2022_29998_MOESM4_ESM.pdf]

Title: Supplementary Data 1
Description: A List of SNPs in the UK IBD cohort B The effect of various SNPs based on the iSNP pipeline

Title: Supplementary Data 2
Description: The Transcription factor target feedback loop of the UCassociated signalling network: a) List of Transcription factors, b) Transcription factortarget gene network, c) Enriched Gene Ontology Biological Processes BenjaminiHochberg corrected Hypergeometric tests

Title: Supplementary Data 3
Description: Enriched Gene Ontologies per clusters and the differentially expressed genes in the transcriptomic validation Benjamini-Hochberg corrected hypergeometric tests

Title: Supplementary Data 4
Description: SNP affected genes in the control networks

Title: Supplementary Data 5
Description: Gene Ontology Biological Processes overrepresentation in the control networks Benjamini-Hochberg corrected hypergeometric tests

Title: Supplementary Data 6
Description: Gene Ontology Biological Processes overrepresented in commonly affected proteins

Title: Supplementary Data 7
Description: SNP affected genes and effecting regulators
