# Supplementary material for: A systems genomics approach to uncover patient-specific pathogenic pathways and proteins in ulcerative colitis
Source: Nat Commun. 2022 Apr 28;13:2299. doi: 10.1038/s41467-022-29998-8 (PMC9051123; doi:10.1038/s41467-022-29998-8)
Supplement: Supplementary file 3 — Peer Review File [file 41467_2022_29998_MOESM3_ESM.pdf]

nature portfolio

Peer Review File

A systems genomics approach to uncover patient-specific pathogenic pathways and proteins in ulcerative colitis

Open Access This file is licensed under a Creative Commons Attribution 4.0 International License, which permits use, sharing, adaptation, distribution and reproduction in any medium or format, as long as you give appropriate credit to the original author(s) and the source, provide a link to the Creative Commons license, and indicate if changes were made. In the cases where the authors are anonymous, such as is the case for the reports of anonymous peer reviewers, author attribution should be to 'Anonymous Referee' followed by a clear attribution to the source work. The images or other third party material in this file are included in the article's Creative Commons license, unless indicated otherwise in a credit line to the material. If material is not included in the article's Creative Commons license and your intended use is not permitted by statutory regulation or exceeds the permitted use, you will need to obtain permission directly from the copyright holder. To view a copy of this license, visit http://creativecommons.org/licenses/by/4.0/.

Reviewers' Comments:

Reviewer #1:
Remarks to the Author:
Brooks et al propose an approach that integrate single nucleotide polymorphism network platform (iSNP), designed to identify the exact mechanisms of how SNPs affect cellular regulatory networks, and how SNP co-occurrences contribute to disease pathogenesis in ulcerative colitis (UC). Using SNP profiles of 377 UC patients, they mapped the regulatory effects of the SNPs to a human signaling network containing protein-protein, miRNA37 mRNA and transcription factor binding interactions. Further, they applied unsupervised clustering algorithms grouped these patient-specific networks into four distinct clusters based on two large disease hubs, NFKB1 and PKCB. The iSNP approach identified regulatory effects of disease-associated non-coding SNPs, and identified how pathogenesis pathways are activated via different genetic modifications. Overall, the approach is reasonable and useful in post-GWAS era to find the role of causative SNPs. The major concern is about how well the iSNP could be generalized and whether we could reproduce similar results in other complex diseases. The second big concern is about the robustness of the approach, There are multi-steps involved in creating the disease-specific network for non-coding SNPs, but authors never check at each step about the robustness of the results.

1. The signaling network consisted of protein-protein, miRNA and transcriptional interactions. In total, the UC-associated signaling network consisted of 247 protein nodes and 1,269 protein-protein interactions, regulated by 4 transcription factors and 25 miRNAs with altogether 682 regulatory interactions. All the results and interpretation are based on this network. But, authors, never test how robust is this UC associated signaling network. For example, Omnipath (Nat Methods. 2016 Nov 29;13(12):966-967) is highly influenced by the literature curation of the interaction from different sources. This might be the reason that NFKB1 and PKCB that are highly studied genes are the hubs in this sub-network. They should test that these genes are not because of the way the network is constructed.
2. The network footprint of each patient contained the proteins encoded by the SNP-affected genes and the interactors of these proteins, i.e. their first neighbor proteins. Why the first neighbors were considered? Authors give an example that first neighbour disease-associated proteins in both diabetes and juvenile idiopathic arthritis, but what is the rationale using here, is it because the network is incomplete or first neighbors transmit the signals?
3. iSNP approach should be tested whether it is robust if we remove random edges from the network.
4. What is the rationale of using GLay clustering method? Will the results change if we apply other clustering methods?

I will stop here, hoping that I've made my point. Without systematic testing of the approach, it is difficult to judge the results in the manuscript.

Reviewer #2:
Remarks to the Author:
Of note, this same manuscript has been pre-published in a non-peer-reviewed form and is already online at (https://www.biorxiv.org/content/10.1101/692269v1.full)

Brooks et al. aimed to build an integrative single nucleotide platform (iSNP) for identifying how non-coding SNPs affect the cellular regulatory networks in complex diseases. The study integrates SNPs (genome) and transcriptome, which are part of the ulcerative colitis (UC) interactome, and the authors applied their new workflow to genomic and transcriptomic UC public data, as well as genomic data from an East Anglian UC cohort (n=377), and used an additional University of Leuven cohort (n=44) of UC patients. Their workflow included integrating independent genomic and transcriptomic data, signaling network analysis, and utilization of the 1st neighbor concept. Although the goals of the study are important and the study is generally well thought and extensive, multiple and important flaws are noted as detailed below, and some degree of overinterpretation is present. A series of concerns, comments and questions are offered to the authors for their consideration.

1. The authors have used multiple prediction tools in their analysis. Yet, they do not provide any justification for choosing—among a number of other available tools—the ones described in the manuscript. For example, for miRNA target sites prediction they used miRanda. Yet, the model driving TargetScan (v7.0 targetscan.org) has been shown to be performing significantly better than other existing models (Agarwal V, Bell GW, Nam JW, Bartel DP. Predicting effective microRNA target sites in mammalian mRNAs. Elife. 2015. doi: 10.7554/eLife.05005. PMID: 26267216; PMCID: PMC4532895). It is advisable to provide justification of their tool choices and confirmation of their predictions using at least another equally or better performing tool.

2. To assess the effects of the SNPs in microRNA-TSs, the authors studied the mature sequence of microRNAs (~22 nucleotides). The authors should consider also studying the pre-miRNA sequences, since SNPs may affect the processing of miRNA production and expression.

3. The authors focused to annotate SNPs in the transcription factor binding sites (TFBS) and in microRNAs. From the 40 UC-identified SNPs, only 12 SNPs localized within the TFBS and microRNAs. Are any of the rest 28 SNPs located in regulatory/promoter areas of coding genes related to UC?

4. The authors identified NFKB1 and PCKB as the two central hubs. However, the GNA12/LSP1 module has 71 proteins, more than NFKB1 (55 proteins) network. What is their justification of choosing NFKB1 relative GNA12/LSP1?

5. Since chromatin modulation seems to be common between clusters 1, 3 and 4, suggesting their importance in UC, the authors should perform an analysis using an HDAC7+ cluster and a HDAC7- cluster unless they consider cluster 4 as the HDAC7+ cluster. Is there any correlation between HDAC7 or LSP1 mRNA levels with UC patient treatment?

6. The results of the GO enrichment analysis appear to add little new to the results of this study (line 216-230). The finding of "645 GO terms that were enriched in at least one patient" is not surprising as the vast majority of them are involved in most biological processes. Furthermore, the choice of "regulation of intracellular signal transduction" and "positive regulation of response to stimulus" as the two GO biological processes defined as "major players" in UC pathogenesis is not well justified given the fact that most other complex diseases would involve the two general GO biological processes. Similar comments are made in regard to the fact that the authors associated 3 of the 4 UC clusters to specific GO biological process terms (line231-240). This lessens the value of the GO analysis and its specific relevance to UC pathogenesis.

7. The definition of "The remaining six non-hub SNP-affected proteins due to their lower number of interactions" seems arbitrary. What does "lower" mean in this context? What was the cut-off number of interactions for "lower"? How was this determined?

8. The section entitled "non-hub SNP-affected proteins influence UC pathogenesis" (line 285-336) is recommended for deletion. Defining 4 UC clusters based on signaling pathways should be enough to fulfill the goals of the study, and adding another section extending the putative regulation of UC to another large sets of proteins adds relatively little value considering that even the 4 UC clusters have not been tested in any biological system. Furthermore, the section is exceedingly speculative and only adds theoretical and putative considerations.

9. How and why was the East Anglian UK cohort, which is part of the larger UK IBD genetics consortium, chosen for this study? The SNP profiling method as well as population structure and a detailed list of the clinical parameters of this cohort should have been provided. The same is true for the description of the patients of the public UC genomic and transcriptomic data used in the analysis.

10. From the 377 UC patients, 246 were on mesalamine treatment and 124 has received additional immunomodulatory therapies. Are there any clusters related to specific immunomodulatory treatments? Any correlation with biologics treatment? The 4 clusters presented correlate with

32/124 patients on immunomodulators. How about the other modules (e.g. LSP1, HDAC7), how do they correlate with these treatments?

11. Did the authors investigate whether any correlation exists between each of the four UC signaling clusters with clinical phenotype of the patients? This is of major importance to determine whether specific (or at least selective) signaling network pathways underlie the various clinical presentations of UC. This would go a long way to better understand and define the biological heterogeneity of this form of IBD. The same is true for the different forms of treatment that the UC patients may have been on.

12. There appears not to be any evidence of input in the manuscript from the clinical IBD experts listed among the co-authors; no information is provided about duration of disease, disease extension, type of therapy, length of therapy, etc. The possible effect of treatment on the expression of the network genes has not been addressed or discussed. This could have allowed an opportunity for a better biological correlation and perhaps a more precise patient clustering, data essential to the understanding of how much heterogeneity exists in this subtype of IBD.

13. To investigate whether non-hub SNP-affected proteins impact on regulation of inflammation in UC the authors analyzed the transcriptome of paired inflamed and non-inflamed colonic biopsies of the 44 patient UC Leuven cohort. They concluded that the SNP profiles of this cohort were similar to the UK IBD cohort, but this is predictable given the fact that both UC cohort have the same basic disease, i.e., chronic intestinal inflammation. Moreover, the conclusion is considerably weakened by the lack of critically important information: 1) how were "inflamed" and "non-inflamed" biopsies classified? By endoscopic appearance only or histological analysis? This is a common flaw in most studies of IBD as it is well established that endoscopically normal mucosa commonly shows inflammation at the histological level; 2) were the clinical characteristics of the East Anglian cohort comparable to that of Leuven in regard to length of disease, extent of colonic inflammation, clinical severity, type of therapy, etc. This does not seem to be the case as the Leuven cohort consisted of patient with severe UC necessitating anti-TNF therapy.

14. The issue of "how SNP co-concurrences contribute to disease pathogenesis in UC" has not been formally tested, even selectively, in any experimental biological system (in vitro or in vivo) in this report.

15. The use of the terminology "patient-specific UC-associated signaling networks or network footprints" is questionable. Does the term "specific" refer to networks detected in each patient in this particular study or "disease specific"? No other inflammatory controls, such as Crohn's disease patients, were used and tested in this study.

16. In the conclusion the authors state that they "identified novel pathways linking the pathogenic effectors of genetic susceptibility, immune modulation and environmental triggers". Which environmental triggers are linked by the results is unclear as also why and how the author reached this conclusion.

17. At line 521 the patients of the East Anglian cohort are stated as "aged between 25 and 100 years". Is this correct? No subject who is 100-year-old should be included in any study except for senescence studies given the known effects of senescence on immunity and inflammation.

18. The Supplementary Material legends need to be more descriptive e.g., what do the colors in Sup Tables 1 and 5 mean?

Response to Reviewers for Manuscript ID: NCOMMS-19-19617A

We thank the reviewers for their feedback of the manuscript and the constructive comments. The comments and questions led to a significant and appropriate revision of the integrated Single nucleotide polymorphism Network Platform (iSNP) workflow and the manuscript.

Reviewer #1

Brooks et al propose an approach that integrate single nucleotide polymorphism network platform (iSNP), designed to identify the exact mechanisms of how SNPs affect cellular regulatory networks, and how SNP co-occurrences contribute to disease pathogenesis in ulcerative colitis (UC). Using SNP profiles of 377 UC patients, they mapped the regulatory effects of the SNPs to a human signaling network containing protein-protein, miRNA37 mRNA and transcription factor binding interactions. Further, they applied unsupervised clustering algorithms grouped these patient-specific networks into four distinct clusters based on two large disease hubs, NFKB1 and PKCB. The iSNP approach identified regulatory effects of disease-associated non-coding SNPs, and identified how pathogenesis pathways are activated via different genetic modifications. Overall, the approach is reasonable and useful in post-GWAS era to find the role of causative SNPs. The major concern is about how well the iSNP could be generalized and whether we could reproduce similar results in other complex diseases. The second big concern is about the robustness of the approach, There are multi-steps involved in creating the disease-specific network for non-coding SNPs, but authors never check at each step about the robustness of the results.

We thank the Reviewer for these general comments - with specific regard to the concerns about reproducibility and generalizability. Ulcerative colitis was used as an example of a complex genetic disease with a high burden of non-coding single nucleotide polymorphisms, which is found in other complex genetic diseases e.g. Type1 diabetes, Atherosclerosis, Rheumatoid arthritis. We felt that bringing in multiple differing disease types would remove clarity from this methodology-focused paper, and would have required so many parallel analysis and subsequent *in vitro* validation via transcriptomics that it would have looked basically like a collection of mini-papers.
The robustness of the approach has been significantly added to the revised version with parameter and cut off analysis (see Methods) as well as additional *in vivo* data based validation steps for each major analysis e.g. SNP affected proteins, patient-specific clusters and affected pathways.

1. The signaling network consisted of protein-protein, miRNA and transcriptional interactions. In total, the UC-associated signaling network consisted of 247 protein nodes and 1,269 protein-protein interactions, regulated by 4 transcription factors and 25 miRNAs with altogether 682 regulatory interactions. All the results and interpretation are based on this network. But, authors, never test how robust is this UC associated signaling network. For example, Omnipath (Nat Methods. 2016 Nov 29;13(12):966-967) is highly influenced by the literature curation of the interaction from different sources. This might be the reason that NFKB1 and PKCB that are highly studied genes are the hubs in this sub-network. They should test that these genes are not because of the way the network is constructed.

We thank the Reviewer for this comment, which we took on board. We reanalysed the network and looked for the effect of curation bias as a possible reason why certain genes became cluster drivin to ensure in the novel analysis the hubs were not just promiscuous proteins with the highest level of curated data within the background network (OmniPath). We have shown that this is not the case - NFKB1 was not a cluster driving protein, and less promiscuous cluster driving proteins were identified e.g. VEGFA/XPO5/POLH and SNAI1/CEBPB/PTPN1. This is documented in the revised manuscript in the 'Identification of patient-specific clusters based on the UC-associated network' section of the Results.

2. The network footprint of each patient contained the proteins encoded by the SNP-affected genes and the interactors of these proteins, i.e. their first neighbor proteins. Why the first neighbors were considered? Authors give an example that first neighbour disease-associated proteins in both diabetes and juvenile idiopathic arthritis, but what is the rationale using here, is it because the network is incomplete or first neighbors transmit the signals?

Very good point that was indeed not clear in the original manuscript. The rationale is as the reviewer mentioned: Without the first neighbours, the network would be incomplete, and we have demonstrated in this and other studies that the first neighbours of disease-associated genes have often a critical role to transmit disease relevant signals. We have added this explanation into the Introduction section.

3. iSNP approach should be tested whether it is robust if we remove random edges from the network.

Thank you for this comment. The network was originally reconstructed using literature curated signaling interactions from OmniPath. To address the Reviewer's comment, we now tested the iSNP workflow and its results with other network resources, such as experimentally validated protein-protein interactions from STRING and signaling interaction curated for Reactome. The results are in the Supplementary Results section of the revised manuscript. In brief, the SNP affected functions were similar in all three tested networks, but OmniPath covered more affected biological processes. The clustering of the patients depended on the network-specific degree of the SNP-affected proteins. In STRING and Reactome the primary driver for clustering were the HLA proteins, meanwhile those were tertiary and quaternary sectors in the patient clustering using OmniPath. We did not use random shuffling of the nodes because if we keep the degree of the nodes, then the same nodes will have high degree. We added further explanation about the sensitivity of the network resource and the degree of the nodes to the Discussion section.

4. What is the rationale of using GLay clustering method? Will the results change if we apply other clustering methods?

We thank the Reviewer for raising this question. GLay clustering via the Clustermaker Cytoscape app was used, as it is optimised for large networks and allows for the analysis of highly connected networks - which our network is. We have added further explanation to the Methods section on the use of the GLay clustering method.

GLay clustering or Louvain clustering is not a deterministic method. This means if we rerun the clustering we do not get exactly the same results. The function of the modules depends mostly on the affected proteins and their topological role within the network. Due to these concerns we have note analysed the module structures extensively. With using two additional control network resources, we were able to show that the affected Gene Ontology Biological Processes are overlapping, independently of the network resources.

We thank the Reviewer for the very useful comments that helped us to improve the iSNP workflow and develop the revised manuscript.

Reviewer #2

Of note, this same manuscript has been pre-published in a non-peer-reviewed form and is already online at (https://www.biorxiv.org/content/10.1101/692269v1.full) Brooks et al. aimed to build an integrative single nucleotide platform (iSNP) for identifying how non-coding SNPs affect the cellular regulatory networks in complex diseases. The study integrates SNPs (genome) and transcriptome, which are part of the ulcerative colitis (UC) interactome, and the authors applied their new workflow to genomic and transcriptomic UC public data, as well as genomic data from an East Anglian UC cohort (n=377), and used an additional University of Leuven cohort (n=44) of UC patients. Their workflow included integrating independent genomic and transcriptomic data, signaling network analysis, and utilization of the 1st neighbor concept. Although the goals of the study are important and the study is generally well thought and extensive, multiple and important flaws are noted as detailed below, and some degree of overinterpretation is present. A series of concerns, comments and questions are offered to the authors for their consideration.

We are grateful for the Reviewer for the detailed review of our manuscript and for these helpful and constructive comments. We have altered the workflow as per the Reviewer's suggestions, reanalysed the data, validated the data using a large transcriptomic dataset and significantly rewritten the manuscript to take your concerns and comments on board.

1. The authors have used multiple prediction tools in their analysis. Yet, they do not provide any justification for choosing—among a number of other available tools—the ones described in the manuscript. For example, for miRNA target sites prediction they used miRanda. Yet, the model driving TargetScan (v7.0 targetscan.org) has been shown to be performing significantly better than other existing models (Agarwal V, Bell GW, Nam JW, Bartel DP. Predicting effective microRNA target sites in mammalian mRNAs. Elife. 2015. doi: 10.7554/eLife.05005. PMID: 26267216; PMCID: PMC4532895). It is advisable to provide justification of their tool choices and confirmation of their predictions using at least another equally or better performing tool.

We thank the Reviewer for this request for clarification and justification for the tools we have used. Accordingly, we have updated the manuscript with the justification in the discussion. We used the miRanda tool because it was able to provide miRNA target sites to any given sequence. Following the Reviewer's suggestion we attempted to implement TargetScan into the iSNP pipeline. However, TargetScan needs genome comparisons that made it hard to integrate to an automatic workflow, and that step was exponentially long. After months of trying

different approaches, including discussing the issue with the TargetScan developers, we had to make a decision that we can't yet integrate TargetScan to iSNP. As mentioned now in the revised Discussion, the iSNP workflow can be updated with other tools when they will be available and compatible with an automated system.

On the other hand, for the transcription factor binding sites, we were able to directly address the Reviewer's valid request, and we integrated two tools, FIMO and RSAT. We extended the Methods section accordingly.

2. To assess the effects of the SNPs in microRNA-TSs, the authors studied the mature sequence of microRNAs (~22 nucleotides). The authors should consider also studying the pre-miRNA sequences, since SNPs may affect the processing of miRNA production and expression.

Whilst this suggestion would be ideal, unfortunately, upon checking it, the available data on pre-miRNA sequences currently disallowed this. We had concerns about adding false-positivity to the network, therefore, we acknowledge this valid suggestion as an area to consider for future study, and added a sentence to the Discussion section.

3. The authors focused to annotate SNPs in the transcription factor binding sites (TFBS) and in microRNAs. From the 40 UC-identified SNPs, only 12 SNPs localized within the TFBS and microRNAs. Are any of the rest 28 SNPs located in regulatory/promoter areas of coding genes related to UC?

We thank the Reviewer for this comment. The rest of the SNPs were not in the promoter regions based on our previous analysis, however we needed to increase the coverage so to do that we updated the iSNP workflow and analysed UC associated SNPs localised not only in promoter regions but also in enhancer regions. This resulted in 20 which are in TFBS. The updated data on our findings is found in Table 1. The number of SNPs that are localised into regulatory areas has increased.

4. The authors identified NFKB1 and PCKB as the two central hubs. However, the GNA12/LSP1 module has 71 proteins, more than NFKB1 (55 proteins) network. What is their justification of choosing NFKB1 relative GNA12/LSP1?

Thanks for requesting this clarification. In the revised manuscript we extended the network, as described above, and re-analysed the data to include all of the points outlined by the reviewers; this all resulted in a change in the original module structure. However, the point raised by the Reviewer is still valid for other modules in the revised work. We ordered the cluster driving proteins based on the number of interactors and which level they influence the hierarchical clustering of the patients. Basically we have chosen those proteins which have more first neighbours. This is documented in the Results section of the manuscript.

5. Since chromatin modulation seems to be common between clusters 1, 3 and 4, suggesting their importance in UC, the authors should perform an analysis using an HDAC7+ cluster and a HDAC7- cluster unless they consider cluster 4 as the HDAC7+ cluster. Is there any correlation between HDAC7 or LSP1 mRNA levels with UC patient treatment?

We thank you for this comment. In the revised and extended work, the HDAC7 cluster was not identified as a cluster driving protein, therefore we did not perform this further analysis.

6. The results of the GO enrichment analysis appear to add little new to the results of this study (line 216-230). The finding of “645 GO terms that were enriched in at least one patient” is not surprising as the vast majority of them are involved in most biological processes. Furthermore, the choice of “regulation of intracellular signal transduction” and “positive regulation of response to stimulus” as the two GO biological processes defined as “major players” in UC pathogenesis is not well justified given the fact that most other complex diseases would involve the two general GO biological processes. Similar comments are made in regard to the fact that the authors associated 3 of the 4 UC clusters to specific GO biological process terms (line231-240). This lessens the value of the GO analysis and its specific relevance to UC pathogenesis.

We thank these comments, which we took on board. We have altered the GO analysis considerably. We analysed the Gene Ontology pathways of cluster driving proteins and compared these to Gene Ontology pathways of the differentially expressed genes from a large transcriptome study using specifically the rectum samples of index colonscopies of patients with ulcerative colitis. By using these methods and validation, we removed the ‘general’, and high level GO biological processes and drilled down to the specific processes involved in UC. For example, calcium homeostasis, wound healing and cellular response to stress, and immune cell motility and cell adhesion. This is documented in the revised manuscript in Figure 3C and Figure 4D.

7. The definition of “The remaining six non-hub SNP-affected proteins due to their lower number of interactions” seems arbitrary. What does “lower” mean in this context? What was the cut-off number of interactions for “lower”? How was this determined?

Thank you for this question. Following comments from other reviewers, this form of analysis no longer appears in the manuscript.

8. The section entitled “non-hub SNP-affected proteins influence UC pathogenesis” (line 285-336) is recommended for deletion. Defining 4 UC clusters based on signaling pathways should be enough to fulfill the goals of the study, and adding another section extending the putative regulation of UC to another large sets of proteins adds relatively little value considering that even the 4 UC clusters have not been tested in any biological system. Furthermore, the section is exceedingly speculative and only adds theoretical and putative considerations.

We agreed with the Reviewer, and removed the section.

9. How and why was the East Anglian UK cohort, which is part of the larger UK IBD genetics consortium, chosen for this study? The SNP profiling method as well as population structure and a detailed list of the clinical parameters of this cohort should have been provided. The same is true for the description of the patients of the public UC genomic and transcriptomic data used in the analysis.

We thank the Reviewer for pointing out that this justification was missing from the original manuscript. We have added to a paragraph with the reasoning for the use of the East Anglian Cohort in the Supplementary discussion of the revised manuscript. Furthermore, the clinical parameters are now included in the supplementary tables. The SNP profiling method is referenced in Jostins et al, but as no further profiling was undertaken, further details have not been included. For the transcriptomic dataset obtained from NCBI GEO, we have correctly referenced it as per the Reviewer's suggestion.

10. From the 377 UC patients, 246 were on mesalamine treatment and 124 has received additional immunomodulatory therapies. Are there any clusters related to specific immunomodulatory treatments? Any correlation with biologics treatment? The 4 clusters presented correlate with 32/124 patients on immunomodulators. How about the other modules (e.g. LSP1, HDAC7), how do they correlate with these treatments?

We analysed if the updated clusters correlate to any clinical parameters. We found no significant differences based on the cohorts (Chi-square tests $p>0.05$, One way ANOVA $p>0.05$, Supplementary Table 3). This is not unexpected as it required nearly 30,000 patients for Cleynen and colleagues (Lancet 387, 156–167 (2016)) to identify NOD2, MHC and 3p21 as being associated with age of disease onset and disease location in IBD. We have updated the discussion of the revised manuscript accordingly.

11. Did the authors investigate whether any correlation exists between each of the four UC signaling clusters with clinical phenotype of the patients? This is of major importance to determine whether specific (or at least selective) signaling network pathways underlie the various clinical presentations of UC. This would go a long way to better understand and define the biological heterogeneity of this form of IBD. The same is true for the different forms of treatment that the UC patients may have been on.

As clusters did not correlate to clinical parameters, we have not taken this further other than to comment that larger datasets such as the UK-based Gut Reactions are needed.

12. There appears not to be any evidence of input in the manuscript from the clinical IBD experts listed among the co-authors; no information is provided about duration of disease, disease extension, type of therapy, length of therapy, etc. The possible effect of treatment on the expression of the network genes has not been addressed or discussed. This could have allowed an opportunity for a better biological correlation and perhaps a more precise patient clustering, data essential to the understanding of how much heterogeneity exists in this subtype of IBD.

We thank the Reviewer for pointing out that the contribution of the clinical co-authors was unclear. To confirm, all clinicians have had input into the design of the workflow and to the original as well as to the revised manuscript. We have updated the authors' contributions section to correctly represent their work.

The patient metadata is held within the supplementary figures, but as no correlation was identified, direct analysis was not taken further, however a paragraph on the effect of gene expression and treatment was added in to the Discussion of the revised manuscript.

13. To investigate whether non-hub SNP-affected proteins impact on regulation of inflammation in UC the authors analyzed the transcriptome of paired inflamed and non-inflamed colonic biopsies of the 44 patient UC Leuven cohort. They concluded that the SNP profiles of this cohort were similar to the UK IBD cohort, but this is predictable given the fact that both UC cohort have the same basic disease, i.e., chronic intestinal inflammation. Moreover, the conclusion is considerably weakened by the lack of critically important information: 1) how were "inflamed" and "non-inflamed" biopsies classified? By endoscopic appearance only or histological analysis? This is a common flaw in most studies of IBD as it is well established that endoscopically normal mucosa commonly shows inflammation at the histological level; 2) were the clinical characteristics of the East Anglian cohort comparable to that of Leuven in regard to length of disease, extent of colonic inflammation, clinical severity, type of therapy, etc. This does not seem to be the case as the Leuven cohort consisted of patient with severe UC necessitating anti-TNF therapy.

We thank this comment. We changed the validation cohort to a larger patient cohort, with samples from an index colonoscopy (rectum) with active UC, to overcome the flaw of microscopic vs macroscopic inflammation.

14. The issue of "how SNP co-concurrences contribute to disease pathogenesis in UC" has not been formally tested, even selectively, in any experimental biological system (in vitro or in vivo) in this report.

We thank the Reviewer for this valid comment. We no longer specifically reference SNP co-concurences, as we appreciated that the original manuscript required further validation analysis, therefore we have focused on three forms of validation of the *in silico* outputs, using UC transcriptome samples. With this we have updated the focus of the manuscript as well.

15. The use of the terminology "patient-specific UC-associated signaling networks or network footprints" is questionable. Does the term "specific" refer to networks detected in each patient in this particular study or "disease specific"? No other inflammatory controls, such as Crohn's disease patients, were used and tested in this study.

We thank the Reviewer for pointing this out. We have changed the terminology accordingly. As the focus of the manuscript is on the workflow and validation of it, using UC as an example, we wished to retain clarity of focus as described in our response to Reviewer 1 (point 1).

16. In the conclusion the authors state that they "identified novel pathways linking the pathogenic effectors of genetic susceptibility, immune modulation and environmental triggers". Which environmental triggers are linked by the results is unclear as also why and how the author reached this conclusion.

Thank you for this comment. We have deleted this section and re-written the discussion to reflect the new outcomes.

17. At line 521 the patients of the East Anglian cohort are stated as “aged between 25 and 100 years”. Is this correct? No subject who is 100-year-old should be included in any study except for senescence studies given the known effects of senescence on immunity and inflammation.

We thank the Reviewer for pointing out that the age of the patients was unclear and misleading. We have adjusted this to reflect the age at which they were in the inclusion of the Immunochip study, not the age at which they were when the data was analysed.

18. The Supplementary Material legends need to be more descriptive e.g., what do the colors in Sup Tables 1 and 5 mean?

The supplementary figures have been updated, and therefore this legend is no longer present.

We are grateful for the suggestions and specific comments of the Reviewer that helped us to improve the manuscript. While the revised work resulted in partially different biological pathways and proteins, the comments of the Reviewer on the original data guided us on how to analyse the new data and we took into account the Reviewer’s previous comments and suggestions.

Reviewers' Comments:

Reviewer #2:
Remarks to the Author:
The authors have addressed all comments in a thorough and satisfactory fashion, and have considerably modified the version of the revised manuscript. All previous concerns have been properly addressed.
